# A Synergistic Hydrogel‐Microalgae Platform for Dual‐Targeting of Intestinal and Neuroimmune Dysfunction in Inflammatory Bowel Disease

**DOI:** 10.1002/advs.202523551

**Published:** 2026-04-14

**Authors:** Jing Lu, Kangyu Jin, Bing Chen, Ruoxi Wang, Fengling Hu, Shaohua Hu, Danni Zhong, Xiaoming Li, Min Zhou

**Affiliations:** ^1^ Department of Psychiatry, The First Affiliated Hospital Zhejiang University School of Medicine Hangzhou China; ^2^ Zhejiang Key Laboratory of Precision Psychiatry Hangzhou China; ^3^ Institute of Translational Medicine Zhejiang University Hangzhou China; ^4^ Department of Gastroenterology, The First Affiliated Hospital Zhejiang University School of Medicine Hangzhou China; ^5^ Department of Neurobiology and Department of Neurology of Second Affiliated Hospital Zhejiang University School of Medicine Hangzhou China; ^6^ Zhejiang University‐Ordos City Etuoke Banner Joint Research Center, Zhejiang University‐University of Edinburgh Institute (ZJU‐UoE Institute), Zhejiang University School of Medicine Zhejiang University Haining China

**Keywords:** cognition, depression, inflammatory bowel disease, microbiota‐gut‐brain axis, paeoniflorin

## Abstract

Inflammatory bowel disease (IBD) is frequently complicated by comorbid depression and anxiety, creating a therapeutic vicious cycle that is currently managed with fragmented, non‐integrated treatments. Here, we introduce a colon‐targeted, pH‐responsive hydrogel microalgal system (CV@PA‐gel) designed for synergistic treatment of IBD and its psychiatric comorbidities. This engineered platform co‐encapsulates the natural neuroprotective agent paeoniflorin (PA) and the gut‐microbiota modulator Chlorella vulgaris (CV) within a genipin‐crosslinked carboxymethyl chitosan/sodium alginate matrix. The CV@PA‐gel exhibits minimal drug release in the stomach but provides sustained, targeted release in the colon, significantly enhancing the oral bioavailability and intestinal retention of its cargo. In a murine model of chronic colitis, CV@PA‐gel outperforms free PA by more effectively restoring gut barrier integrity, ameliorating systemic and hippocampal inflammation, and rescuing anxiety‐, depressive‐like, and cognitive behaviors. Mechanistically, our findings suggest that gut‐derived systemic inflammation is associated with complement C3 activation and subsequent microglia‐mediated polarization of neurotoxic A1 astrocytes in the hippocampus, leading to synaptic loss. PA, delivered precisely by the hydrogel, directly suppresses this cascade by inhibiting microglial release of key A1‐inducing factors. Our work establishes a versatile biomaterials strategy for disrupting the gut‐brain axis pathology, offering a powerful platform for the simultaneous management of intestinal and neuropsychiatric disorders.

## Graphical abstract

A bioactive hydrogel incorporating Chlorella vulgaris and paeoniflorin within a carboxymethyl chitosan/sodium alginate matrix cross‐linked with genipin is developed for inflammatory bowel disease therapy. The hydrogel exhibits gastric stability and intestine‐responsive release. Oral treatment restores the intestinal barrier, alleviates gut dysbiosis, and ultimately alleviates symptoms of colitis‐associated depression and cognitive impairment.

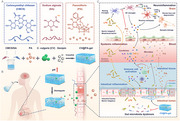

## 1 Introduction

Inflammatory bowel disease (IBD) is a group of chronic intestinal inflammatory disorders [1]. Individuals with IBD frequently suffer from depression, anxiety, and cognitive impairments [2, 3, 4], which can significantly compromise their quality of life, complicate clinical management [5], and potentially exacerbate the frequency of disease activity [6]. Current clinical approaches remain largely fragmented: only 2% of adult IBD patients receive adequate psychological support, and most antidepressant treatments are administered for durations shorter than recommended guidelines [7]. This highlights a critical unmet need for integrated therapeutic strategies that concurrently address both intestinal inflammation and its associated neuropsychiatric manifestations.

The microbiota‐gut‐brain axis provides a conceptual framework for understanding how intestinal inflammation communicates with the brain to influence behavior and cognition [8]. Key mediators of this communication include a leaky intestinal barrier, which permits the translocation of bacterial endotoxins like lipopolysaccharide (LPS) into systemic circulation, triggering widespread inflammation [9]. While these pathways are increasingly recognized, the precise molecular mechanisms linking peripheral immune activation to specific neural pathologies in IBD remain elusive. Emerging evidence positions the complement system, an ancient arm of innate immunity, as a potential linchpin [10]. Complement component C3, in particular, is a known mediator of pathological synaptic pruning in depression and is reported to be elevated in IBD patients [11, 12, 13]. We thus hypothesized that gut inflammation‐driven systemic endotoxemia could activate hippocampal complement C3 signaling, potentially driving microglia‐dependent polarization of neurotoxic A1 astrocytes [14], thereby providing a mechanistic basis for the comorbid cognitive‐affective impairments.

Addressing this complex pathophysiology requires an agent with dual anti‐inflammatory and neuroprotective properties. Paeoniflorin (PA), a bioactive monoterpene glycoside, emerges as a compelling candidate [15]. It is known to block macrophage release of complement C1q and improve intestinal inflammation [16], and demonstrates efficacy in models of depression [17, 18, 19]. However, its clinical translation is severely hampered by formidable pharmacokinetic challenges, including poor oral bioavailability and rapid systemic clearance [20]. This delivery bottleneck necessitates an innovative formulation strategy to unlock its full therapeutic potential.

To overcome these limitations, we devised a biomaterials‐based strategy centered on a colon‐targeted, synergistic delivery system. We report the design of a pH‐responsive hydrogel, CV@PA‐gel, for the co‐delivery of PA and *Chlorella vulgaris* (CV), a probiotic microalga with known gut‐barrier protective and microbiota‐modulating properties [21]. This system was engineered by encapsulating PA and CV within a genipin‐crosslinked matrix of carboxymethyl chitosan and sodium alginate (CMCS/SA). The resulting hydrogel is designed to remain intact in the harsh acidic environment of the stomach, minimizing premature drug release, and to swell and degrade in the neutral pH of the colon, ensuring targeted payload delivery and enhanced local retention.

**SCHEME 1:**
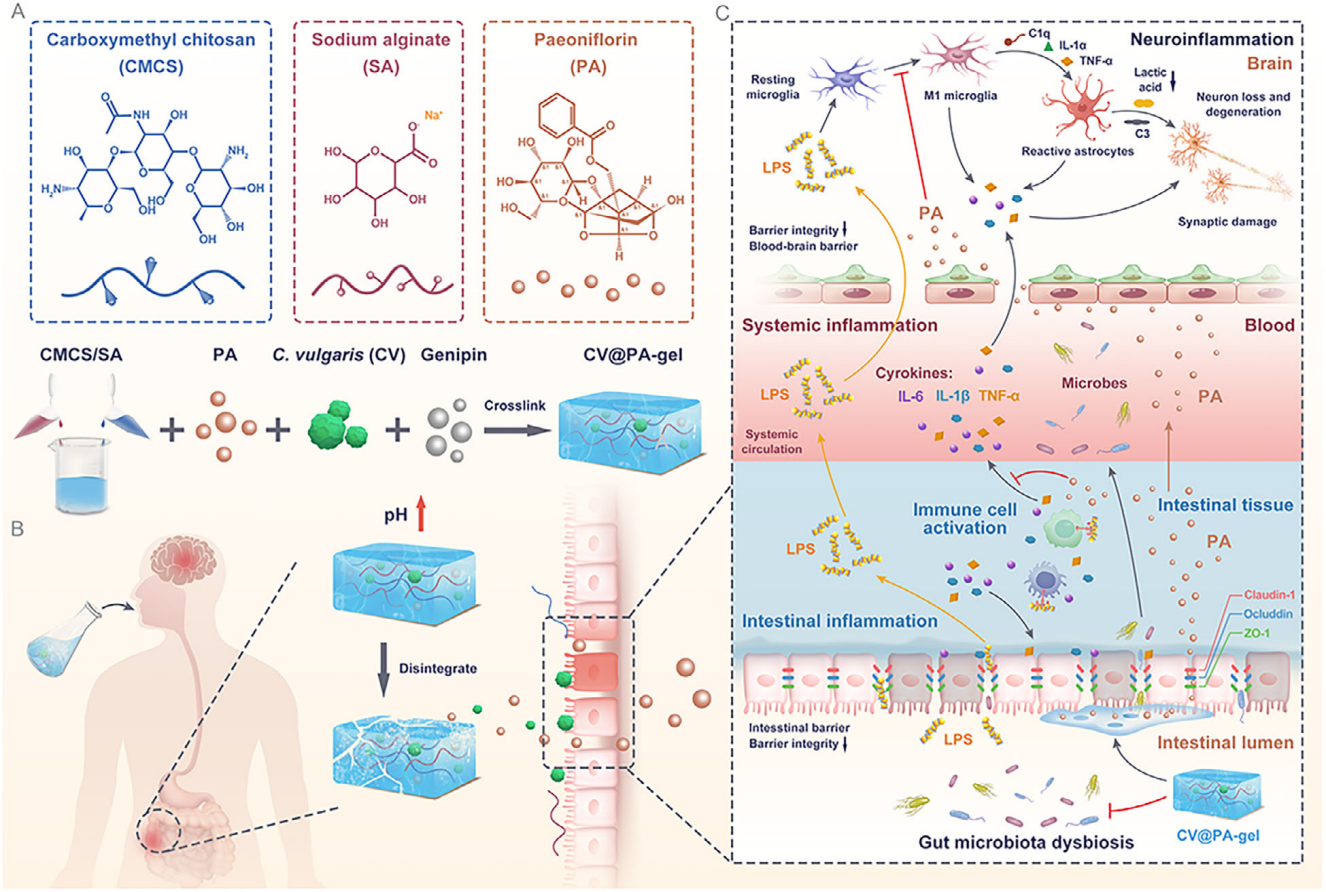
Bioactive hydrogel loaded with microalgae and herbal constituent for gut‐brain axis regulation in inflammatory bowel diseases. (A) Chlorella vulgaris (CV) and paeoniflorin (PA) were co‐encapsulated with carboxymethyl chitosan (CMCS)/sodium alginate (SA) mixture and cross‐linked with genipin to form bioactive hydrogels (CV@PA‐gel). (B) CV@PA‐gel showed good gastric stability, prolonged intestinal retention time and intestine‐responsive release. (C) Oral treatment of CV@PA‐gel improved the intestinal barrier by reducing the inflammatory response and alleviating intestinal flora and metabolic imbalances and partially alleviated brain inflammation in IBD mice via the microbial‐gut‐brain axis. In addition, oral administration of CV@PA‐gel directly blocked the generation of A1 astrocytes induced by microglia activation, restored the chronic DSS‐induced impairment of neurogenesis and synaptic plasticity, and improved cognitive ability.

Herein, we present this engineered CV@PA‐gel system as a versatile platform for treating IBD and its psychiatric comorbidities (Scheme 1). We first validate our scientific hypothesis by demonstrating that chronic colitis indeed triggers a C3‐driven A1 astrocytic response in the hippocampus, concomitant with synaptic and behavioral deficits. We then elucidate the direct cellular target of PA, showing it abrogates microglial induction of A1 astrocytes. With this mechanistic foundation established, we comprehensively characterize our biomaterial, confirming its pH‐responsive drug release profile and enhanced gastrointestinal retention. Finally, we demonstrate the superior therapeutic efficacy of CV@PA‐gel over free PA in a chronic colitis model, showcasing its unique ability to concurrently ameliorate intestinal pathology, normalize systemic and neuroinflammation, and robustly rescue associated anxiety‐, depressive‐like, and cognitive behaviors. Distinct from existing PA delivery systems or probiotic formulations, CV@PA‐gel combines a pH‐responsive hydrogel matrix with a living microalgal component to achieve colon‐targeted delivery, improved drug retention, and microbiota modulation, thereby enabling an integrated biomaterials strategy to intervene in gut‐brain axis pathology. Our work transcends conventional monotherapies by providing a synergistic, biomaterial‐driven platform that effectively disrupts the vicious cycle of gut‐brain inflammation, offering a novel and integrated therapeutic paradigm.

## 2 Results

### 2.1 Endotoxin Leakage and Complement–Glial Activation in IBD Patients

**FIGURE 1:**
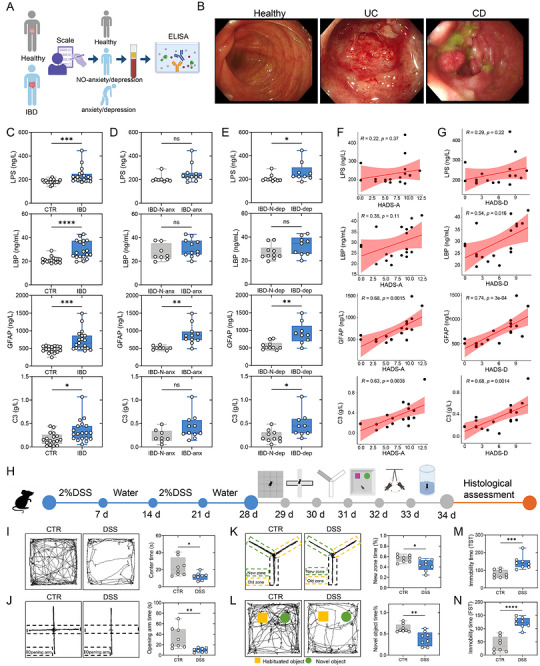
General characteristics and cytokine characteristics of clinical subjects and DSS‐induced chronic colitis mouse with anxiety, depressive‐like behaviors. (A) Clinical study cohorts. (B) Representative endoscopic images of healthy and IBD patients. (C) Changes of Lipopolysaccharide (LPS), Lipopolysaccharide binding protein (LBP), Glial fibrillary acidic protein (GFAP) and Complement Component 3 (C3) in healthy controls and inflammatory bowel disease (IBD) patients (n = 17, 19). (D, E) Changes of those molecular in IBD without or with anxiety (D) (n = 8, 11) and depression (E) (n = 10, 9). (F) Correlation between plasma level LPS, LBP, GFAP, C3 with anxiety scores from HADS scales in IBD patients. (G) Correlations between plasma level LPS, LBP, GFAP, C3 with depressive scores from HADS scales in IBD patients. (H) Schematic illustration of the construction of DSS‐induced chronic colitis mice model: mice were treated with two cycles of DSS (1 week 2% DSS water followed by 1 week water per cycle) and evaluate colitis‐associated anxiety, depressive‐like behaviors, and cognitive behaviors. (I–N) Center time in the open field test (I), opening arm time in the elevated plus maze (J), the proportion of time exploring in the new zone in the Y maze (K), in novel object in the novel object recognition test (L) and the immobility time in tail suspension test (TST) (M) and forced swimming test (FST) (N), respectively (n = 8). The significance of difference of LPS, LBP and GFAP in (C), LPS and LBP in (D, E) was determined by Mann‐Whitney test, while the significance of difference of C3 in (C), GFAP and C3 in (D, E) and in (I–N) was determined by an unpaired t‐test. ns, no significance *p* ≥ 0.05; *p < 0.05; **p < 0.01; ***p < 0.001; ****p < 0.0001.

To establish the link between intestinal inflammation and neuropsychiatric symptoms, we first analyzed a cohort of IBD patients and healthy controls (Figure 1A,B). Active IBD patients exhibited a high prevalence of comorbid depression (47%) and anxiety (58%) (Table S1). Consistent with gut barrier dysfunction, plasma levels of LPS and LBP were significantly elevated compared to controls (Figure 1C). Notably, these patients also showed increased levels of the neuroinflammatory markers GFAP and complement C3, which correlated strongly with the severity of depressive and anxiety symptoms (Figure 1D–G), suggesting a potential role for endotoxin‐driven complement activation in neuropsychiatric comorbidity.

To mechanistically dissect this link, we employed a murine model of chronic DSS‐induced colitis (Figure 1H). DSS‐treated mice recapitulated key clinical features, including colonic inflammation, impaired intestinal barrier (Figure S1A–C), and significant anxiety‐like, depressive‐like, and cognitive deficits (Figure 1I–N). Crucially, this model revealed a cascade from systemic inflammation to hippocampal pathology: increased circulating and hippocampal LBP and C3 (Figure S1D–F) were accompanied by robust neuroinflammation, complement C3 activation, and a shift in astrocytes toward a neurotoxic A1 phenotype (Figure S1G–L). This glial dysregulation was associated with significant synaptic loss in the hippocampus (Figure S1M–Q), suggesting a potential mechanism for the observed behavioral impairments.

### 2.2 Paeoniflorin Targets Microglia to Suppress A1 Astrocyte Polarization

We next sought a therapeutic agent capable of disrupting this C3‐driven glial cascade. Screening of eight anti‐inflammatory compounds identified paeoniflorin (PA) as a potent inhibitor of key A1‐inducing factors (*Il1a*, *Tnfa*, *C1qa*) (Figure S2A–C, Table S2) [20, 22, 23, 24, 25, 26, 27, 28, 29, 30, 31]. Molecular docking indicated stable binding of PA to these cytokines (Figure S2D). We further validated the neuroprotective effect of PA in a mixed glial‐PC12 co‐culture model, where it significantly attenuated LPS‐induced impairment of neurite outgrowth and branching (Figure S2E–H). To definitively establish the cellular target of PA, we employed a conditioned medium approach using primary microglia and astrocytes [32]. We found that PA pretreatment of LPS‐stimulated primary microglia abolished their capacity to induce A1 astrocyte polarization, as evidenced by a reduction in C3d+/GFAP+ cells (Figures S2I, J). Furthermore, PA restored the metabolic function of astrocytes [33, 34, 35], which had been impaired by activated microglial conditioned medium, by reversing deficits in basal glycolysis and compensatory glycolytic capacity (Figure S2K–M). Consequently, neurons exposed to astrocyte‐conditioned medium derived from PA‐treated microglia exhibited preserved morphology and reduced apoptosis (Figure S3A–C). Critically, PA exerted no direct effect on astrocytes or neurons (Figure S2J and S3D,E), underscoring that its protective effects are exclusively mediated via the inhibition of microglial activation. These results establish PA as a promising neuroprotective agent that acts by specifically inhibiting microglia‐dependent A1 astrocytic polarization.

### 2.3 Engineering CV@PA‐Gel to Surpass the Limitations of PA

**FIGURE 2:**
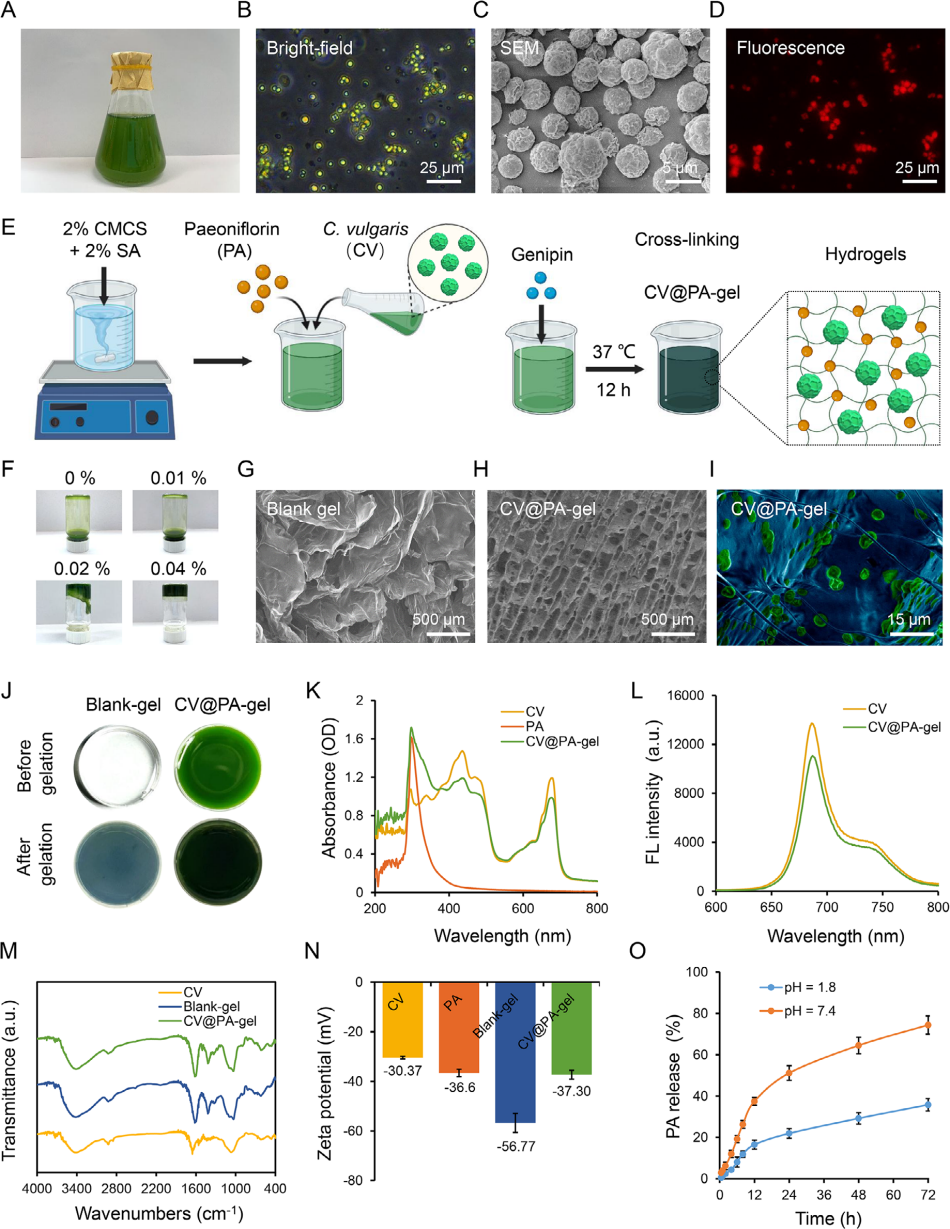
Synthesis and characterization of CV@PA‐gel. (A) Photograph of the large‐scale culture of CV under laboratory conditions. (B) Bright‐field microscope images of CV. Scale bar, 25 µm. (C) SEM image of CV. Scale bar, 5 µm. (D) Fluorescence microscope images of CV. Scale bars, 25 µm. (E) Synthetic route of CV@PA‐gel via adding paeoniflorin and CV to CMCS/SA mixture and cross‐linking with genipin. (F) Photographs of CV@PA‐gel formed at different final concentrations of genipin (0, 0.01, 0.02, and 0.04%). (G) SEM image of Blank‐gel. Scale bar, 500 µm. (H) Low‐magnification and (I) High‐magnification SEM images of CV@PA‐gel. The scale bars are 500 and 15 µm for low and high, respectively. (J) Photographs of Blank‐gel and CV@PA‐gel before and after gelation. (K) UV–vis absorption spectra of CV, PA, and CV@PA‐gel. (L) Fluorescence emission spectra of CV and CV@PA‐gel under the excitation wavelength of 552 nm. (M) FTIR spectra of CV, PA, and CV@PA‐gel. (N) Zeta potential of CV, PA, Blank‐gel and CV@PA‐gel. (O) Drug release behavior of CV@PA‐gel at different pH conditions (pH = 7.4 and 1.8). Data are presented as means ± SD. Data are representative of three independent experiments. SEM, scanning electron microscope; CMCS, carboxymethyl chitosan; SA, sodium alginate; UV–vis, ultraviolet and visible spectroscopy; FTIR, Fourier transform infrared spectroscopy.

Despite its therapeutic potential, the clinical translation of PA is limited by poor oral bioavailability and rapid clearance. To overcome this delivery bottleneck, we employed a hydrogel strategy that enables controlled release of PA in response to intestinal conditions and facilitates prolonged retention within the intestines. CV, as a natural dietary supplement, could synergistically modulate the gut microbiota composition and function (Figure S4A) [36, 37, 38]. The green‐colored CV cells exhibit sizes ranging from approximately 2 to 3 µm (Figure 2A–C) and display strong red fluorescence under appropriate laser irradiation (Figure 2D).

A pH‐sensitive hydrogel (CV@PA‐gel) composed of carboxymethyl chitosan/sodium alginate (CMCS/SA) was utilized for co‐delivery of CV and PA to improve intestinal retention and targeting efficacy upon oral administration (Figure 2E). Hydrogel formation by CV@PA‐gel was unsuccessful at genipin concentrations below 0.02% (Figure 2F; Figure S4B). Taking into account both gelation and fluidity, the synthesis condition involving a final genipin concentration of 0.2% was chosen for subsequent experiments. CMCS/SA blank hydrogel (Blank‐gel) were both dense and porous (Figure 2G). while CV@PA‐gel displayed numerous CV cells (green) attached to the surface or dispersed within the hydrogel pores (Figure 2H–J). Furthermore, UV–vis, fluorescence emission, and FTIR spectra confirmed characteristic peaks corresponding to CV, PA, and BLANK‐gel in CV@PA‐gel (Figure 2K–M). The incorporation of both CV and PA partially masked the negative charge of CV@PA‐gel (Figure 2N). Two simulated environments were utilized: pH 1.8 PBS to mimic the gastric environment and pH 7.4 PBS to simulate the intestinal environment. The release of PA from CV@PA‐gel was found to be slow in the simulated stomach environment, with a 72‐hour release rate of only 35.81%. Conversely, in the simulated intestinal environment, PA was rapidly released from CV@PA‐gel, with a 72‐hour release rate of 74.38% (Figure 2O). In addition, the incorporation of microalgae did not significantly affect the release kinetics of PA (Figure S4D). These pH‐responsive drug release properties of CV@PA‐gel contribute to the reduction of CV and PA loss in the stomach and facilitate targeted release in the intestine, thereby enhancing the bioavailability of CV and PA after oral administration.

### 2.4 Tissue Distribution and Biodegradation After Oral Administration

**FIGURE 3:**
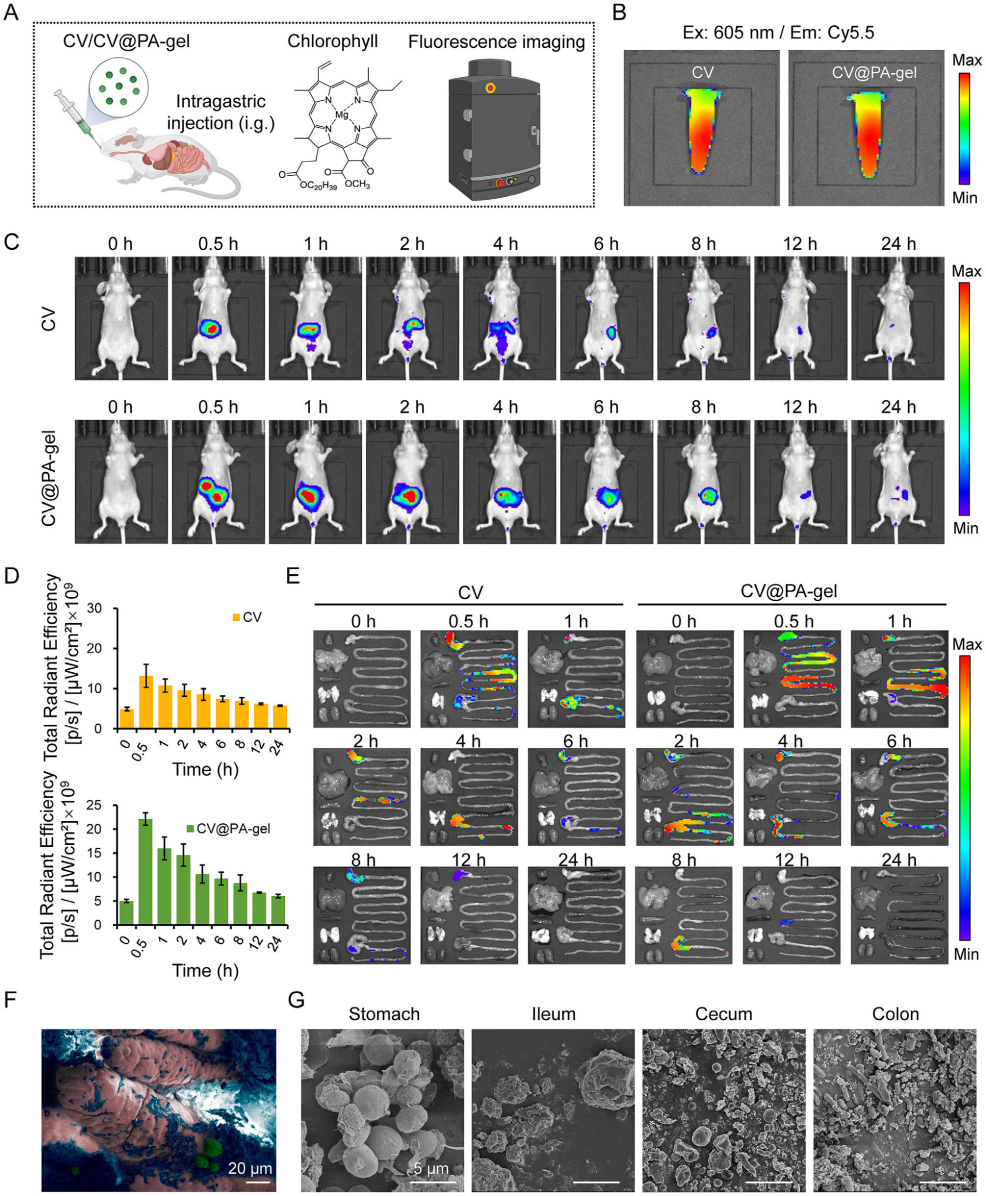
Tissue distribution and biodegradation of CV@PA‐gel. (A) Schematic illustration of chlorophyll‐based fluorescence imaging for investigating the biodistribution of CV and CV@PA‐gel after intragastric administration. (B) In vitro fluorescence imaging of CV and CV@PA‐gel samples under the chlorophyll fluorescence channel (Ex: 605 nm; Em: Cy5.5). (C) Time‐dependent in vivo fluorescence imaging of mice after intragastric administration with 300 µL of CV (2.8 × 10^7^ cells/mL) and CV@PA‐gel (CV = 2.8 × 10^7^ cells/mL, PA = 4 mg/mL). (D) Total radiant efficiency of mice at different time points (n = 3). (E) Time‐dependent ex vivo fluorescence imaging of different tissues (heart, liver, spleen, lung, kidney, and gastrointestinal tract) of mice after intragastric administration with 300 µL of CV (2.8 × 10^7^ cells/mL) and CV@PA‐gel (CV = 2.8 × 10^7^ cells/mL, PA = 4 mg/mL). (F–G) SEM images of the gastrointestinal (stomach, ileum, cecum, and colon) contents of mice after intragastric administration with CV@PA‐gel (CV = 2.8 × 10^7^ cells/mL, PA = 4 mg/mL). Scale bars, 5 µm.

Notably, the chlorophyll‐based in vivo fluorescence imaging and distribution of CV@PA‐gel after oral administration were assessed, a discernible fluorescent signal was observed in the abdominal region of the mice, which gradually descended and diminished over a period of time (Figure 3A–C). The fluorescence signal suggesting the potential of hydrogels in facilitating drug adhesion and prolonging the retention time within the gastrointestinal system (Figure 3D,E). Notably, no fluorescence signal was detected in the major organs of mice, implying that the metabolism of CV@PA‐gel primarily occurred within the gastrointestinal tract. (Figure 3F,G). Overall, CV@PA‐gel exhibited remarkable in vivo fluorescence imaging capabilities, as well as favorable intestinal retention and biodegradability.

### 2.5 Evaluation of the Biocompatibility and Oral Biosafety of CV@PA‐Gel

**FIGURE 4:**
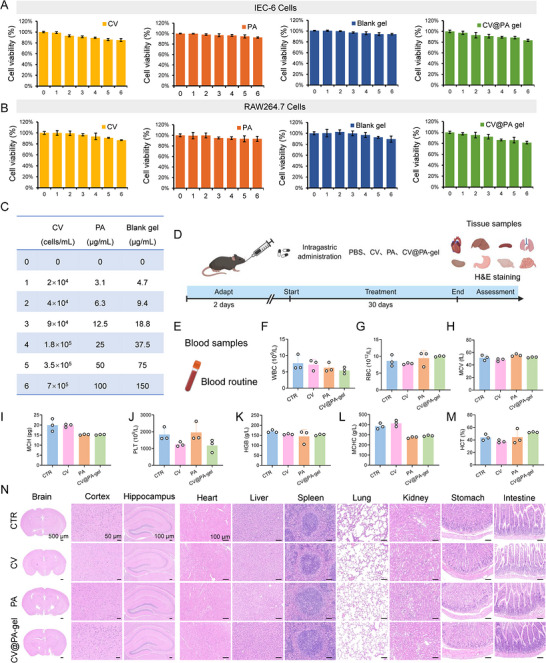
In vitro cytocompatibility and oral safety evaluation of CV@PA‐gel. (A‐B) Cell viabilities of IEC‐6 cells (A) and RAW264.7 cells (B) after co‐culture with different concentrations of CV, PA, Blank‐gel, and CV@PA‐gel for 24 h (n = 3). (C) The different concentration of CV, PA and Blank‐gel. (D) Schematic illustration of biosafety assessment. Intragastric interventions (saline, CV, PA, or CV@PA‐gel) were performed daily for 30 consecutive days following a 2‐day adaptation period. After treatment, the whole blood and serum of mice were collected for blood routine (n = 3) and biochemical analysis (n = 3), and the major organs (brain, heart, liver, spleen, lung, kidney, stomach and intestine) were excised for histopathological examination. (E–M) Blood routine and biochemical analysis of mice after different treatments (n = 3). Data are presented as means ± SD. (N) H&E staining of brain heart, liver, spleen, lung, kidney, stomach and intestine tissues of mice after different treatments. Scale bars, 500, 50 and 100 µm, respectively.

The in vitro biocompatibility of CV@PA‐gel was systematically assessed in IEC‐6 and RAW264.7 cells. Treatment with CV, PA, Blank‐gel, or CV@PA‐gel across a broad range of concentrations did not cause noticeable cytotoxicity, with cell viability maintained above 90%, indicating excellent cytocompatibility (Figure 4A–C). Both PA and CV@PA‐gel demonstrated significant reduction in LPS‐induced ROS elevation in two different cell cultures (Figure S5A–D). To further examine the long‐term in vivo biosafety, we evaluated the long‐term oral biosafety of CV@PA‐gel for up to 30 days. (Figure 4D). Hematological analyses showed no significant differences among different groups, suggesting normal systemic physiological conditions (Figure 4E–M). Consistently, histopathological examination revealed intact tissue morphology and the absence of inflammatory infiltration or structural damage in the brain (including the cortex and hippocampus), heart, liver, spleen, lung, kidney, stomach, and intestine (Figure 4N). These findings demonstrate that prolonged oral administration of CV@PA‐gel does not induce systemic toxicity or local tissue damage, confirming its favorable biocompatibility and biosafety for subsequent therapeutic use.

### 2.6 Systemic Endotoxemia Drives Complement‐Mediated Neuropsychiatric Deficits

To directly validate the causal sufficiency of endotoxemia in mediating behavioral deficits, we employed a chronic low‐dose LPS exposure model involving a 0.5 mg/kg intraperitoneal injection administered over 10 consecutive days (Figure S6A). Mice subjected to LPS exposure exhibited anxiety‐like behavior, evidenced by reduced time spent in the center during the OFT and decreased exploration of the opening arms in the EPM (Figure S6B, C); depressive‐like phenotypes, indicated by prolonged immobility in both the TST and the FST (Figure S6F, G); and cognitive impairment, as manifested by diminished preference for new arms in the Y‐maze and reduced novel object recognition in the NOR (Figure S6D, E). Importantly, this intervention resulted in a significant increase in circulating C3 (Figure S6H), mirroring the systemic complement signature observed in both DSS‐induced colitis and IBD patients. These findings suggest that elevated peripheral endotoxin alone is sufficient to drive C3‐mediated neuropsychiatric morbidity, thereby positioning the LPS‐complement axis as a fundamental mechanism of disease.

### 2.7 CV@PA‐Gel Alleviates Gut Integrity and Psychiatric‐Like Behaviors

**FIGURE 5:**
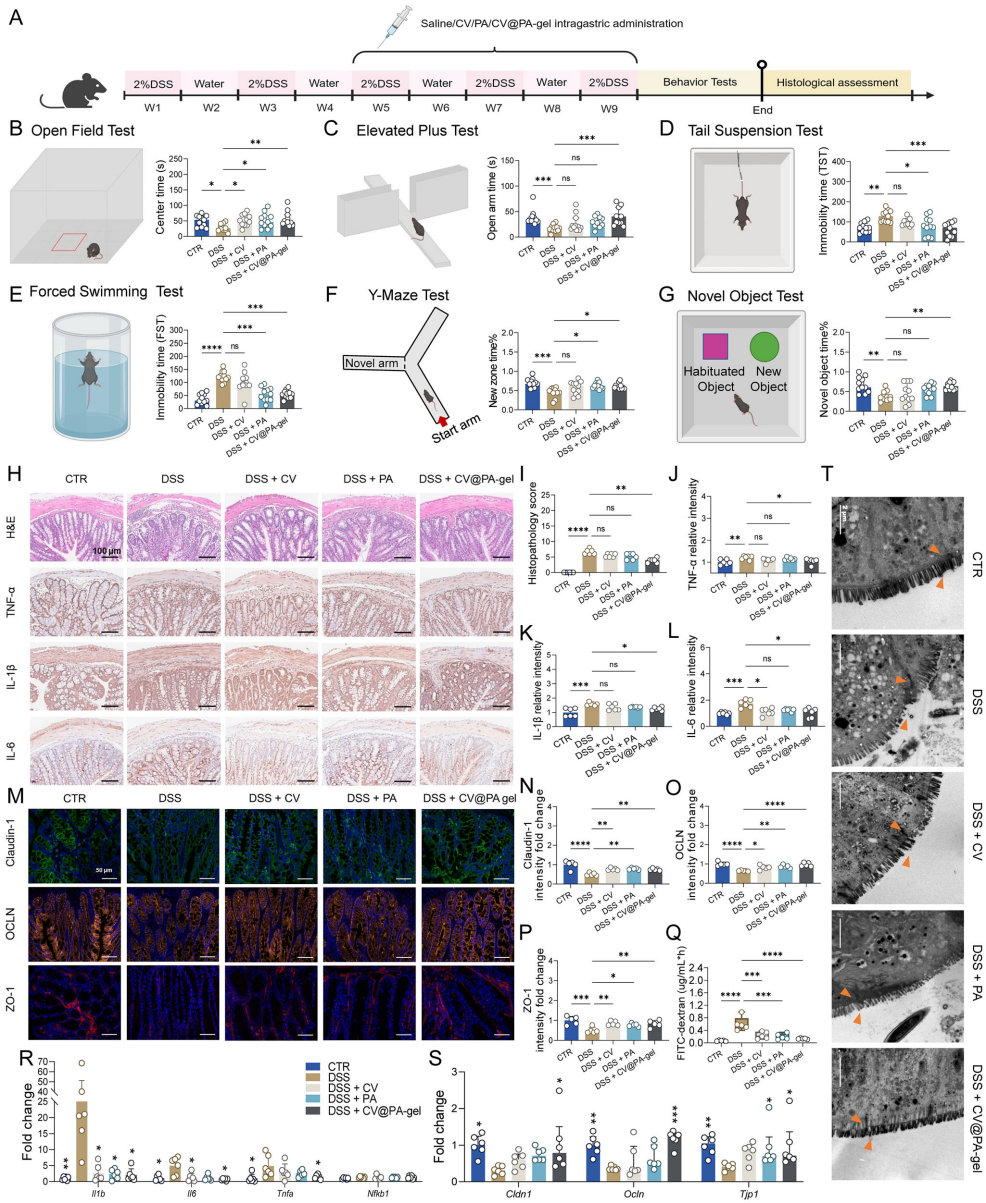
Alleviation effects of CV@PA‐gel on DSS‐induced depressive, anxiety, and cognitive repairment behavioral changes, and inflammation on the intestinal barrier. (A) Schematic of the experimental design. Chronic colitis was induced using a 5‐cycle DSS regimen: mice received 2% DSS in drinking water for 1 week (W1, W3, W5, W7, and W9), interleaved with 1‐week water recovery periods (W2, W4, W6, and W8). Intragastric interventions (saline, CV, PA, or CV@PA‐gel) began at the start of the third DSS cycle (W5) and were continued through the end of the fifth DSS cycle (W9). Behavioral tests were performed after completion of the final DSS cycle, followed by histological assessment. (B–G) Center time in the open field test (B), opening arm time in the elevated plus maze (C), the immobility time in tail suspension test (D) and forced swimming test (E) the proportion of time exploring in the new zone in the Y maze (F) and in novel object in the novel object recognition test (G), respectively (n = 11). (H–L) Representative images (H) and quantification results (I–L) of H&E and immunohistochemical staining (TNF‐α, IL‐1β, and IL‐6) of colon tissues in different groups (n = 6). Scale bars, 100 µm. (M‐P) Representative images (M) and mean immunofluorescence intensity of Claudin‐1 (N), OCLN (O) and ZO‐1 (P) in different groups (n = 6). Scale bars, 100 µm. (Q) FITC‐labeled Dextran (FD4) showed intestinal permeability in different mice (n = 6). (O) mRNA expressions of proinflammatory cytokine (*Il1b*, *Il6*, *Tnfa* and *Nfkb1*) in colon tissues (n = 6). (S) mRNA expressions of *intestinal barrier‐related markers* (*Cldn1*, *Ocln* and *Tjp1*) in colon tissues (n = 6). (T) Representative electron micrographs showing the synaptic structure and postsynaptic densities on neurons (arrowheads point to postsynaptic density) Scale bars, 2 µm. The significance of difference of (B‐G), (I), (K, L), *Il1b* and *Il6* in (R) and *Tjp1* in (S) was determined by Kruskal‐Wallis test, while the significance of difference of (J), (N–Q) *Tnfa* and *Nfkb1* in (R), *Cldn1* and *Ocln* in (S) was determined by one‐way ANOVA with Dunnett's post hoc test. ns, no significance *p* ≥ 0.05; *p < 0.05; **p < 0.01; ***p < 0.001; ****p < 0.0001.

A series of behavioral tests were conducted to assess anxiety, depressive‐like behaviors, and cognitive‐related behaviors in mice (Figure 5A). CV@PA‐gel treatment significantly alleviated anxiety‐like behavior, as evidenced by an increased duration of time spent in the center during the OFT and increased opening‐arms exploration time in the EPM (Figure 5B,C). Additionally, depressive‐like behaviors were rescued, indicated by a decrease in immobility time during both the TST and the FST (Figure 5D,E). Moreover, spatial and recognition memory improved, as demonstrated by performance in the Y‐maze and NOR (Figure 5F,G). These findings suggest that CV@PA‐gel administration effectively mitigates anxiety, depressive‐like behaviors, and cognitive dysfunction in DSS mice.

Concurrently, CV@PA‐gel treatment significantly reduced DSS‐induced colonic inflammation, as evidenced by a reduced incidence of bloody stools (Figure S7A, Table S3), recovery of body weight (Figure S7B), amelioration of colonic shortening (Figure S7C, D), and normalization of splenomegaly (Figure S7E, F). These results were further supported by decreased pathology scores and downregulation of colonic pro‐inflammatory cytokines, including TNF‐α, IL‐1β, and IL‐6 (Figure 5H–L), similar to those of RT‐qPCR results (Figure 5R). The simultaneous restoration of intestinal barrier function was demonstrated by upregulation of tight junction proteins (Claudin‐1, OCLN, and ZO‐1; Figure 5M–P,S), reduced FITC‐dextran permeability (Figure 5Q), and transmission electron microscopy (TEM)‐confirmed ultrastructural repair of epithelial junctions and microvilli (Figure 5T). Validation through in vitro Caco‐2 and RAW264.7 co‐cultures confirmed barrier recovery, evidenced by restored trans‐epithelial electrical resistance (TEER; Figure S7G, H) and reduced paracellular permeability (Figure S7I).

### 2.8 CV@PA‐Gel Inhibits Microglia‐Driven A1 Astrocyte Activation and Preserves Hippocampal Neurons

**FIGURE 6:**
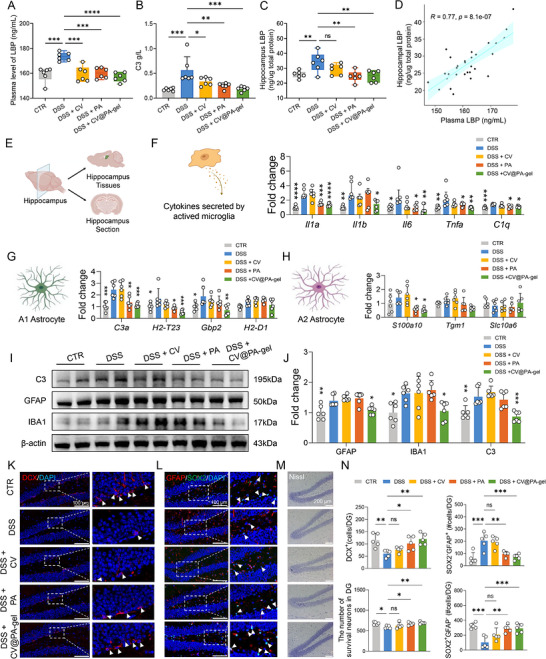
Effects of CV@PA‐gel on cytokines and neuroinflammatory responses related to molecules. (A, B) Plasma lipopolysaccharide‐binding protein (LBP) and C3 (B) changes in different groups (n = 6). (C) Hippocampus LBP changes in different groups of mice (n = 6). (D) Correlations between plasma LBP and hippocampus LBP. (E) Schematic diagram of the hippocampus‐related experimental design in mice. (F) The cytokines secreted by active microglia‐mRNA expressions in the hippocampus (n = 6). (G, H) mRNA expressions of A1 (*C3a*, *H2‐T23*, *Gbp2* and *H2‐D1*) (G) and A2 (*S100a10*, *Tgm1* and *Slc10a6*) (H) astrocyte markers in hippocampus in different groups (n = 6). (I, J) Representative protein bans (I) and quantifications (J) of glia‐related protein (C3, GFAP and IBA1) levels in hippocampus in different groups (n = 6). (K–N) Representative microscopic fields of DCX+ cells (K), GFAP+SOX2+ cells (L), the survival neurons (M) and the quantification results (N) in the DG of the hippocampus in different groups (n = 5). Scale bars, 100 and 200 µm, respectively. The significance of difference of (A‐C), (F‐G), *Tgm1* and *Slc10a6* in (F), (J) and (N) (K, L) was determined by one‐way ANOVA with Dunnett's post hoc test while the significance of difference of *S100a10* in (H) was determined by Kruskal‐Wallis test. ns, no significance *p* ≥ 0.05; *p < 0.05; **p < 0.01; ***p < 0.001; ****p < 0.0001.

Concurrent with the restoration of intestinal barrier integrity, CV@PA‐gel also mitigated neuroinflammation by modulating the activation of A1 astrocytes. Plasma analyses revealed that DSS‐induced activation of complement C3 and endotoxemia, indicated by LBP, were both normalized by CV@PA‐gel administration (Figure 6A,B). Notably, hippocampal pathology mirrored systemic inflammation, with elevated LBP levels that strongly correlated with plasma concentrations (*R* = 0.77, Figure 6C,D), suggesting peripheral LPS may translocate to the central nervous system. Further analyses of the hippocampus demonstrated that CV@PA‐gel blocked the complement‐dependent neurotoxic pathway (Figure 6E,F). This suppression of upstream signaling molecules directly attenuated the polarization of astrocytes towards the neurotoxic A1 phenotype, as confirmed by the coordinated decrease in mRNA expression of A1‐specific markers (*C3a* and *H2‐T23*), while there was no significant change in the protective A2 marker (Figure 6G,H). Consistently, CV@PA‐gel partially reversed the DSS‐induced upregulation of C3 and GFAP protein levels (Figure 6I,J). Collectively, these findings indicate that CV@PA‐gel specifically inhibits complement signaling and A1 astrocyte activation.

**FIGURE 7:**
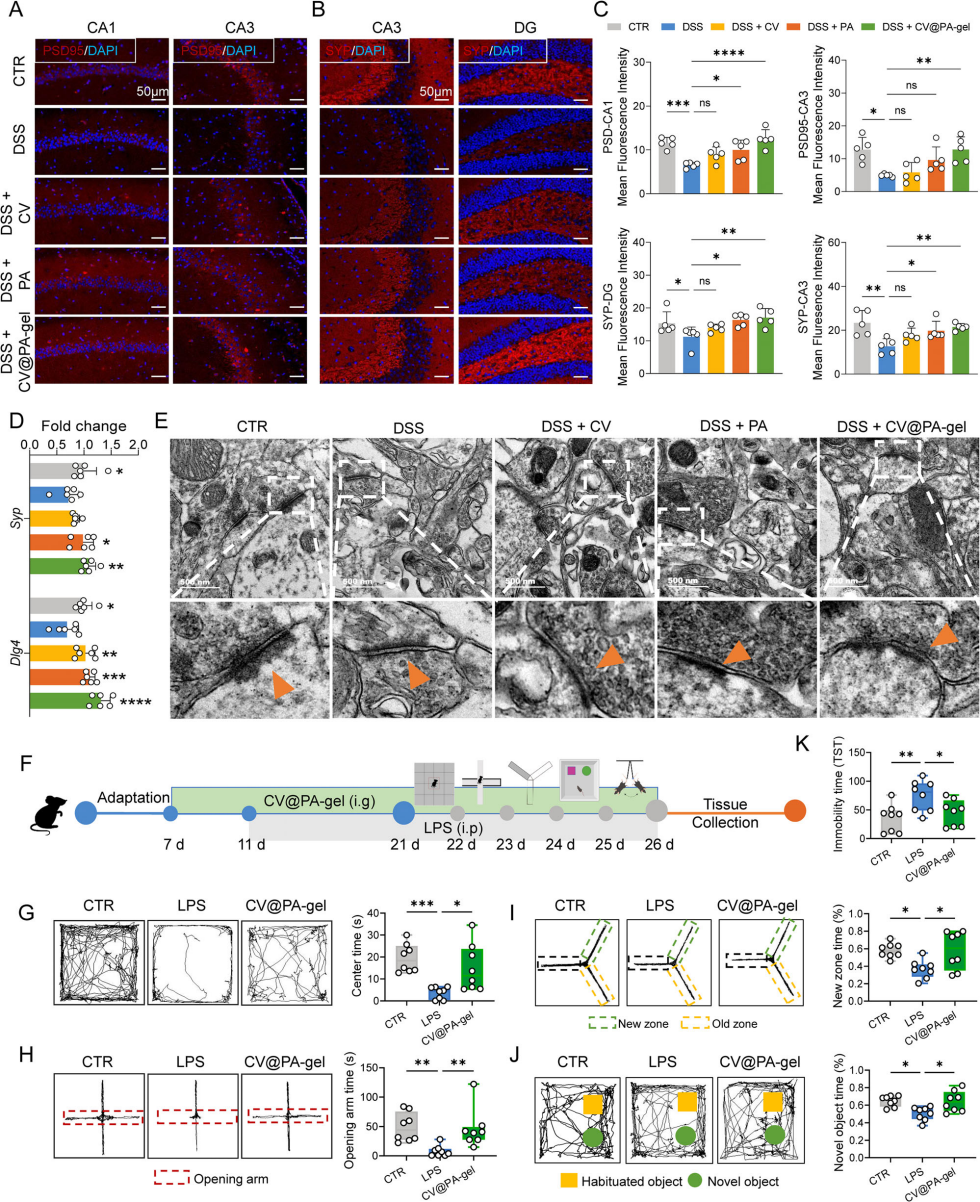
The restorative effects of CV@PA‐gel on the hippocampus. (A–C) Representative microscopic fields of PSD95 (A), SYP (B) and quantifications of mean intensity of fluorescence (C) in the hippocampus in different groups (n = 5). Scale bar, 50 µm. (D) mRNA expression of the synapse‐related proteins (SYP and PSD‐95) in the hippocampus in different groups (n = 6). (E) Representative TEM images of postsynaptic density in the hippocampus in different groups. Scale bar, 500 nm. (F) Schematic illustration of the construction of an LPS‐induced (from day 11) depressed model treated with CV@PA‐gel (from day7) and the behavior tests. (G–K) The center time in open field test (G), opening arm time in elevated plus maze (H), new zone time in Y‐Maze (I), novel object time in novel object recognition test (J) and immobility time in tail suspension test (K) (n = 8). The significance of difference of (C, D) and (K) was determined by one‐way ANOVA with Dunnett's post hoc test while the significance of difference of (G–J) was determined by Kruskal‐Wallis test. ns, no significance *p* ≥ 0.05; *p < 0.05; **p < 0.01; ***p < 0.001; ****p < 0.0001.

Subsequently, we examined the impact of DSS‐induced glial dysfunction on hippocampal neurogenesis and synaptic plasticity. Chronic DSS exposure markedly impaired hippocampal neurogenesis, evidenced by a significant reduction in the number of doublecortin‐positive (DCX^+^) cells, a decline in neural stem and progenitor cells (NSPCs, identified as SOX2^+^GFAP^−^ cells) [39], and a decrease in Nissl‐stained viable neurons within the DG of the hippocampus compared to the CTR (Figure 6K–N). However, administration of CV@PA‐gel partially reversed these deficits (Figure 6K–N), thereby enhancing the regenerative potential of the hippocampus. Additionally, synaptic plasticity significantly contributes to neuroplasticity [40]. Concurrently, synaptic integrity was severely disrupted in DSS‐treated mice, as evidenced by significantly reduced fluorescence intensity and mRNA expression of the synaptic‐associated protein synaptophysin (SYP) and postsynaptic density 95 (PSD‐95) (Figure 7A–D) and thinner postsynaptic densities observed via TEM (Figure 7E). Remarkably, CV@PA‐gel administration restored these alterations, preserving both synaptic structure and function (Figure 7A–E). Furthermore, CV@PA‐gel effectively counteracted the DSS‐induced reduction in levels of brain‐derived neurotrophic factor (BDNF), a key factor closely associated with neuroplasticity (Figure S7J). Collectively, these data confirm that PA prevents microglia‐mediated induction of neurotoxic A1 astrocytes, thereby protecting neurons and preserving synaptic function in the hippocampus.

### 2.9 Regulation on Gut Microbiota and Microbial Metabolites

Beyond its neuroprotective effects on hippocampal synaptic plasticity and neurogenesis, CV@PA‐gel exhibited substantial therapeutic efficacy on the gut ecosystem by functionally modulating gut microbiota composition and metabolic pathways. Administration of CV@PA‐gel significantly increased bacterial alpha‐diversity, effectively mitigating the dysbiosis induced by DSS colitis (Figure S8A, B). Principal coordinates analysis (PCoA) demonstrated a clear shift in the overall gut microbial profile toward a state resembling that of healthy controls, indicating robust restoration of gut microbiota structure (Figure S8C). At the genus level, CV@PA‐gel exerted specific modulatory effects, significantly enriching common beneficial genera such as *Bifidobacterium* and *Lachnoclostridium* while suppressing potentially detrimental ones like *Kocuria* (Figure S8D, E). We also found that CV@PA‐gel improved two DSS depleted bacteria, *Dialister* and *Dorea*, which have been reported to be associated with depression (Figure S8D, E) [41, 42]. Complementary non‐targeted LC‐MS metabolomics revealed CV@PA‐gel's functional impact on host‐microbiome interactions, inducing significant alterations in the fecal metabolomic profile (Figure S8F) and primarily modulating key metabolites, including azelaic acid, 3‐indoleacetic acid and tryptophan (Figure S8G). Dysregulation of these metabolites has been implicated in depression and cognitive dysfunction. Spearman correlation analysis further established significant associations between CV@PA‐gel‐modulated microbial genera and beneficial metabolites, underscoring the integrated gut function restored by this therapeutic formulation (Figure S8H).

### 2.10 CV@PA‐Gel Outperforms in Alleviating Neuropsychiatric Deficits

Neuroinflammation is widely implicated in the pathogenesis of depression and cognitive impairment. In order to account for the potential confounding influence of gut validation improvement on psychiatric symptoms, we employed a chronic intraperitoneal injection of LPS‐induced depression model, to simulate the therapeutic effect of CV@PA‐gel on depression (Figure 7F). Notably, prophylactic treatment with CV@PA‐gel significantly attenuated anxiety, depressive‐like behaviors, and cognitive dysfunction induced by chronic LPS exposure (Figure 7G–K). This result confirms that CV@PA‐gel possesses robust, direct neuromodulatory properties that are independent of its gut‐healing effects.

## 3 Discussion

In conclusion, our preclinical and clinical results identified a subset of IBD with anxiety, depressive‐like behaviors and cognitive dysfunction. We showed that pH‐responsive hydrogels (CV@PA‐gel) can reduce the loss of loaded CV and PA in the stomach acid environment, improve the release kinetics and retention time in the intestine, and thus improve the oral availability of PA. Oral administration of CV@PA‐gel maintains intestinal microecological balance and barrier function, inhibits inflammation and the increase of circulating endotoxin, and prevents the decline of hippocampal neuroplasticity and behavioral defects. Furthermore, the protective effect of CV@PA‐gel is unrelated to its potential impact on neurons but appears to be primarily involved in inhibiting microglia‐induced toxic A1 astrocytes. We found that the chronic enteritis model significantly damaged the intestinal barrier, leading to increased entry of LPS and other components into the body, and some pro‐inflammatory components may enter the brain. Consequently, CV@PA‐gel emerges as a viable and efficacious therapeutic option for individuals with IBD accompanied by anxiety, depression, and cognitive impairment.

The elevation of LPS and LBP caused by intestinal leakage may be the key mediators of the bacteria‐gut‐brain axis connection. Furthermore, there is a notable increase in GFAP levels in IBD patients, which demonstrates a significant positive correlation with the severity of anxiety and depression. Elevated serum GFAP levels may be associated with increased reactive astrocytes and neurological impairment [43]. Clinical studies have reported that GFAP is associated with cognitive impairment and depression [44]. C3 is a key component of the three complement pathways, and imbalances in the complement cascade also trigger proinflammatory responses, manifested in the central system by microglia increasing C3 and neurotoxins through C1q, IL‐1α, or TNF‐α‐mediated astrogliosis [32], which mediate inflammation associated with synaptic and neuronal loss and cognitive dysfunction. Our results indicate that there is an imbalance in the complement cascade in IBD patients, especially in those with anxiety, depression. We also demonstrated the abnormal increase of A1 astrocytes and the therapeutic effect of PA in a mouse model of chronic enteritis. Moreover, the observed protective effect of PA was found to be independent of its potential influence on neurons, but rather attributed to its ability to inhibit the toxic A1 astrocytes induced by microglia. Our findings revealed that the chronic colitis model resulted in substantial impairment of the intestinal barrier, consequently facilitating the entry of LPS and other pathogens into the systemic circulation, some of which may further infiltrate the brain. These pro‐inflammatory constituents triggered the activation of microglia in the hippocampus, exhibiting a characteristic “M1” phenotype. The observed “M1” phenotype aligned with prior findings indicating that LPS triggers microglial activation, and was also in line with the presence of activated microglia in inflammatory models such as depression and DSS‐induced colitis models [45, 46]. Subsequently, this M1 microglial activation prompted the conversion of astrocytes into toxic A1 astrocytes, resulting in the demise of hippocampal neurons and primary hippocampal neurons, as well as impaired neurogenesis and synaptic plasticity in the colitis model. Previous studies, consistent with our findings, have demonstrated that PA can effectively decrease the secretion of C1q in macrophages, which is a crucial factor in the activation of A1 astrocytes [16]. Additionally, PA has been shown to possess protective properties in models of Alzheimer's disease, vascular dementia, and Parkinson's disease [47]. Furthermore, PA may also have therapeutic effects on depression and colitis. Our findings indicated that the primary mechanism of action of PA involves the inhibition of microglia‐induced A1 astrocytes. Intriguingly, within the classical depressive mouse model, the activated microglia were found to exhibit the expression of pivotal factors accountable for the conversion of astrocytes into detrimental A1 astrocytes. This observation aligns with our findings, which indicate that PA effectively impedes the generation of A1 astrocytes. Consequently, PA possesses the potential to confer extensive neuroprotective attributes across a diverse range of neuroinflammatory disorders and nerve impairments characterized by A1 astrocyte activation.

The CV@PA‐gel is demonstrated to be capable of mitigating the degradation of loaded drugs and bioactive substances in the acidic stomach environment, enhancing their release kinetics and retention time in the intestine, and consequently improving the oral therapeutic effects. Previous research has also documented the anti‐inflammatory properties of CV extracts on human intestinal epithelial Caco‐2 cells and DSS‐induced colitis [48]. Our study further highlighted the impact of CV biomass itself on the composition and function of the gut microbiota. It was plausible that CV influenced histidine metabolism and histamine production, thereby safeguarding the integrity of the intestinal barrier. Additionally, our findings indicated that CV exhibited certain anti‐anxiety and anti‐depressive effects, potentially attributable to its ability to modulate gut microbiota and mitigate the entry of pathogenic factors into the brain.

Several limitations of this study should be considered. First, the relatively modest size of the clinical cohort restricts stratification by disease subtype or medications that may affect C3/GFAP levels. Second, while our data reveal a strong association between complement C3 activation, A1 astrocyte polarization, and behavioral deficits in both IBD patients and the colitis models, we did not employ complement‐specific blockade tools (e.g., C3 knockout mice or C3a receptor antagonists) to establish direct causality. Therefore, although our findings are mechanistically consistent with a central role for C3 in mediating gut‐brain axis pathology, the causal relationship remains to be definitively proven. Third, although PA potently inhibits microglial C1q/TNF‐α/IL‐1α production, its precise intracellular targets remain to be fully elucidated. Fourth, while the DSS model effectively recapitulates gut‐brain inflammation, it only partially reflects the chronic neuroplastic changes observed in human IBD. Fifth, the dose dependency of PA's neuroprotective effects has not been systematically evaluated across varying gradients of blood‐brain barrier penetration. Addressing these gaps will require validation in multicenter cohorts and investigations employing conditional glial knockout models and targeted complement inhibition.

## 4 Conclusions

In conclusion, colitis‐induced intestinal barrier dysfunction and gut microbiota migration could potentially lead to complement system damage and excessive growth of reactive astrocytes, contributing to the development of anxiety and depression. Our research provides evidence supporting that CV@PA‐gel treatment, a novel, safe, and effective intervention, can maintain the intestinal barrier and reduce intestinal microbiota translocation, further block microglia‐induced A1 astrocyte transformation, ultimately improved depressive‐like behaviors and cognitive impairment induced by DSS. These findings further suggesting oral administration of CV@PA‐gel may be a potential approach for addressing IBD and comorbid psychiatric disorders by regulating microbiota‐gut‐brain axis, as a promising therapeutic target.

## 5 Experimental Section

### 5.1 Study Cohorts

Patients with active IBD were recruited from the gastroenterology department inpatient unit at First Affiliated Hospital of Zhejiang University School of Medicine (NO. IIT20230361B‐R1), and conducted in accordance with the Declaration of Helsinki. All participants gave written informed consent after a thorough explanation of the study protocol.

### 5.2 Animal

Briefly, C57BL/6J mice received 5 cycles of 2% DSS drinking to induce chronic colitis, and these were treated with PBS, CV, PA, and CV@PA‐gel during the treatment period. After the second cycles, the mice were intragastrically administrated with 300 µL of PBS, PA (4 mg/mL), CV (2.8 × 10^7^ cells/mL) and CV@PA‐gel (CV = 2.8 × 10^7^ cells/mL, PA = 4 mg/mL) every day. All animal experiments were performed according to protocols approved by the local ethics committee and the laboratory animal administration rules of China (2023‐1024).

### 5.3 Statistical Analysis

The data were analyzed using GraphPad Prism software (version 9.3.1, GraphPad Software). Data were presented as means ± SDs for normally distributed variables or as medians ± quartiles for nonnormally distributed variables. Shapiro–Wilk test was used to determine the normal distribution. Mann–Whitney U test was used to determine nonnormal distribution variables between the two groups, and differences among the multigroup were tested with Kruskal‐Wallis (K‐W). And t‐test and one‐way ANOVA were used to determine significant differences in normal distribution variables. Dunnett's t‐test was used for post hoc analysis. Pearson and Spearman correlation index was used to test the correlation between the two groups. Not significant (ns) p ≥ 0.05, *p < 0.05, **p < 0.01, ***p < 0.001 and ****p < 0.0001.

## Author Contributions

Jing Lu, Kangyu Jin, and Bing Chen contributed equally to the work and share first authorship, and the first‐listed name was rotated across academic presentations and the published manuscript, and, accordingly, Jing Lu, Kangyu Jin, and Bing Chen agree and assert that any permutation of the order of these names were corrected and acceptable. conceptualization: J.L., K.J.; data curation: J.L., K.J., D.Z., R.W.; funding acquisition: J.L., M.Z., S.H.; investigation: J.L., K.J., B.C., F.H., D.Z., R.W.; visualization: J.L., K.J., B.C., D.Z.; supervision: X.L., M.Z.; writing – original draft: J.L., K.J.; writing – review & editing: D.Z., R.W.

## Ethics Statement

Patients with active IBD were recruited from the gastroenterology department inpatient unit at First Affiliated Hospital of Zhejiang University School of Medicine (NO. IIT20230361B‐R1), and conducted in accordance with the Declaration of Helsinki. All animal experiments were performed according to protocols approved by the local ethics committee and the laboratory animal administration rules of China (2023‐1024).

## Conflicts of Interest

The authors declare no conflict of interest.

## Supporting information




**Supporting File**: advs75162‐sup‐0001‐SuppMat.pdf.

## Data Availability

The data that support the findings of this study are available from the corresponding author upon reasonable request.
