# Supplementary material for: A Synergistic Hydrogel‐Microalgae Platform for Dual‐Targeting of Intestinal and Neuroimmune Dysfunction in Inflammatory Bowel Disease
Source: Adv Sci (Weinh). 2026 Apr 14;13(36):e23551. doi: 10.1002/advs.202523551 (PMC13317580; doi:10.1002/advs.202523551)
Supplement: Supplementary file 1 — Supporting File: advs75162‐sup‐0001‐SuppMat.pdf. [file ADVS-13-e23551-s001.pdf]

A Synergistic Hydrogel-Microalgae Platform for Dual-Targeting of Intestinal and Neuroimmune Dysfunction in Inflammatory Bowel Disease

Jing Lu. et al.

Corresponding author: Danni Zhong, 11718308@zju.edu.cn; Min Zhou, zhoum@zju.edu.cn

The PDF file includes:

Supplementary methods

Figures S1 to S9

Tables S1 and S5

Supplementary methods

Study cohorts

Participants (n =20) aged 18–65 years were recruited from the First Affiliated Hospital of Zhejiang University School of Medicine. The study was approved by the ethics review boards of the First Affiliated Hospital of Zhejiang University School of Medicine. Inflammatory Bowel Disease (IBD) patients presented at the inpatient department of gastroenterology, and screened using Hospital Anxiety and Depression Scale (HADS) questionnaire, and those who had either depression or anxiety scores over eight points with IBD symptoms were defined as depression and anxiety, respectively. HADS is a self-report questionnaire consisting of two subscales: anxiety (HADS-A, 7 items) and depression (HADS-D, 7 items). Each item is scored on a 4-point scale (0-3), yielding a subscale score range of 0-21, with higher scores indicating greater symptom severity. Consistent with commonly used thresholds, a subscale score ≥8 was used as the cut-off to define clinically relevant anxiety (HADS-A ≥8) and/or depression (HADS-D ≥8) in IBD patients.

Patients with active IBD were recruited from the gastroenterology department inpatient unit at First Affiliated Hospital of Zhejiang University School of Medicine (NO. IIT20230361B-R1), and conducted in accordance with the Declaration of Helsinki. All participants gave written informed consent after a thorough explanation of the study protocol. Active disease was defined by the presence of intestinal inflammation, determined by endoscopy, MRI, At the time of recruitment, we collected blood as well as information on clinical disease activity (Harvey Bradshaw Index or partial Mayo Score, respectively), and patients completed the questionnaires regarding depression and anxiety.

Healthy controls (n=20) matched in age, sex, education and marriage without IBD or any mental disorders were recruited from the medical examination center, and only those with HADS less than five were included.

Among the 40 participants, one patient was excluded due to haemolysis of the

blood sample, two controls were excluded as they declined blood collection, and one control was excluded resulting from later diagnosed with rheumatoid arthritis.

Plasma collection

Plasma cytokine levels detection approximately 3 to 5 mL of blood was aseptically collected from each subject using an approved standard procedure. Subsequently, the blood samples were centrifuged at 3000 rpm for 15 min, separated, and stored at -80°C before analysis.

Animal

Dextran sulfate sodium (DSS)-induced colitis model can mimic some key disease features of human IBD and is the most commonly used model in preclinical studies. We further established the DSS-induced chronic colitis mouse model to investigate the therapeutic effects of CV@PA-gel on IBD and IBD-associated psychiatric disorders, such as anxiety, depression, and cognitive impairment. Briefly, C57BL/6J mice received 5 cycles of 2% DSS drinking to induce chronic colitis, and these were treated with PBS, CV, PA, and CV@PA-gel during the treatment period. After the second cycles, the mice were intragastrically administrated with 300 μL of PBS, PA (4 mg/mL), CV (2.8×10^7 cells/mL) and CV@PA-gel (CV = 2.8×10^7 cells/mL, PA = 4 mg/mL) everyday, N=11 per group. Mice in the control group were fed with autoclaved water during the whole period, and were intragastrically administrated with PBS during the corresponding administration period. At the end of treatment in the tenth week, mice were euthanized for histological assessment after behavioral tests. All the animals were acclimatized for seven days under standard conditions (temperature 22 ± 2 °C and humidity 50%–60% on a 12 h light/dark cycle).

The control group received drinking water without DSS supplementation and was treated with PBS during the treatment period. After treatment, a series of

technique processes, including behavior tests, histological and biochemical analyses were used to evaluate the psychiatric symptoms and intestinal inflammation of mice in different groups. All animal experiments were performed according to protocols approved by the local ethics committee and the laboratory animal administration rules of China (2023-1024).After behaviour test, Mice were anesthetized with pentobarbital and euthanasia.

LPS-induced mouse model: Male C57BL6/J mice (8 weeks) were acclimated for 11 days and injected daily intraperitoneally with LPS (Sigma) dissolved in sterile 0.9% saline at a dose of 0.5mg/kg. This dose is used to stimulate infection without causing significant inflammation or other diseases. Recognizing the potential delayed onset of therapeutic effects associated with oral administration, we proactively administered CV@PA-gel to the treatment group via gavage four days prior to the lps intervention. After 10 days, behavioral tests were performed. Twenty-four hours after behavioral testing, brain tissue and plasma were dissected/sampled.

Depressive and cognitive repairment-related behavior

The behavior test was performed after 24-hours adaptation in the room of the behavior test. Open field test (OFT), Elevated Plus Maze Test (EPM), Tail suspension test (TST) and forced swim test (FST) were used to assess depression and anxiety-related behaviors. Y maze test and novel object recognition were used to assess cognition function. For all the behavior tests, each mouse was returned to the cage and again given 75% alcohol to eliminate the experiment region before the test of the next mouse. Behavior tests were conducted in the following sequence: OFT, EPM, Y maze, Novel object recognition, TST and FST, to minimize stress interference, a 24-hour interval was maintained between each test.

Open Field Test

The mice were placed in the center square of the customized wooden box and their activities were observed within 5 min. After the mice adapted to the laboratory for 60 min, the mice were placed in the center of the open field box (40 cm×40 cm). The area 20 cm×20 cm near the center point was set as the central area, and the total distance and residence time of the central area of the mice within 5 min were recorded.

Elevated Plus Maze Test

The Elevated Plus Maze (EPM) has a pair of open arms and a pair of closed arms. Rodents tend to move in the closed arm, but will move in the open arm out of curiosity and inquiry. The EPM test performed 5 min in total, the mice were placed in the crossing of open and closed arm. The time and distance in the open arm were recorded.

Tail Suspension Test

The Tail Suspension Test (TST) is a widely used behavioral test for depressive-like behavior. By observing the mice's struggle behavior while suspended, the researchers were able to assess depressive symptoms in the mice. The head of the experimental mice was suspended downward by attaching the tail, and a desperate immobile state was recorded.

Forced Swim Test

The forced swim test (FST) is the major test for depressive-like behavior. In simple terms, mice were placed in clear acrylic buckets (40 cm high pure water, 25±1°C) for 6 min. A timer was used to record each rat's immobility duration during the last 4 minutes. The water was replaced after each test. Each mouse’s immobility was defined as it floated in the water without struggling or with only slight movements to keep its nose above the water. The test was recorded without the observer knowing the experimental group.

Y-maze test

Y-maze is mainly used to study the spatial working memory of mice, which is

completely based on the nature of experimental rodents to explore new environments. The three arms of Y-maze are randomly assigned: the novel arm (new zone), the start arm, and the other arm (old zone). The novel arm: closed during the training period and open during the test period; the start arm: the arm in which the mouse enters the maze. At first, one arm was randomly closed, and the mice were allowed to explore freely in the remaining two arms for 10 min. After 4 h, the baffle was opened, and each mouse explored freely for 5 min. The time of the mice entering each arm were recorded. The specific calculation formula is: new zone time (%) = [new zone time / (new zone time + old zone time)] × 100%.

Novel object recognition

The novel object recognition experiment is a sophisticated, sensitive behavioral method that uses rodents' innate inclination to approach and explore novel objects to detect animal recognition memory. The basic procedure of a new object recognition experiment consists of two stages: familiarization phase and test phase. The most basic indicator of new object recognition experiments is the time (s) that an animal spends exploring two objects during the test phase. The preference of experimental animals for novel objects can be quantified by the index of the recognition, which is calculated by the exploration time of novel objects and the exploration time of familiar objects during the test period. The specific calculation formula is: Novel object %=(N)/(N+F) ×100 %, where "N" represents the time spent exploring novel objects during the test period, "F" represents the time spent exploring familiar objects during the test period, and the Novel object % takes into account the different levels of exploration activity between animals.

Materials

Chlorella vulgaris (CV) and BG11 medium were purchased from Guangyu Biological Technology (Shanghai, China). Carboxymethyl chitosan (CMCS,

carboxylation> 80%) and Sodium alginate (SA) was purchased from Shanghai Macklin Biochemical Co., Ltd (Shanghai, China). Genipin was purchased from Shanghai Aladdin Biochemical Technology Co., Ltd (Shanghai, China). Paeoniflorin (purity >90%) was purchased from Nantong FeiYu Biological Technology Co., LTD (Nantong, China). De-ionized water (18.2 MΩ/cm) was prepared by a Milli-Q purification system (St. Louis, MO, USA) and used in all experiments. All chemicals and reagents were used without further purification.

Drug release study

The drug release of CV@PA-gel was evaluated by the dialysis bag method. 5 mL of CV@PA-gel was placed in a dialysis bag with a molecular weight cutoff of 3 kDa. The dialysis bag was soaked in 200 mL of PBS with different pH values (1.8 or 7.4) and stirred continuously at 37°C. At different time points (0.5, 1, 2, 4, 6, 8, 12, 24, 48 and 72 h), a certain volume of release medium was taken out to calculate the drug release rate, and replaced with an equal volume of fresh PBS. The paeoniflorin release was analyzed using a UV-2600 spectrophotometer at 413 nm combined with the standard curve of paeoniflorin in PBS. According to the paeoniflorin release at different time points, the cumulative paeoniflorin release rate was calculated.

CCK8 assay

Mouse mononuclear macrophage leukemia cells (RAW264.7) and rat small intestinal epithelial cell line (IEC-6) were used in the cell experiments. RAW264.7 cells were cultured in DMEM containing 10% FBS and 1% penicillin and 1% streptomycin. IEC-6 cells were cultured in DMEM supplemented with 10% FBS, 1% penicillin and 1% streptomycin, and 0.1 U/mL bovine insulin. RAW264.7 and IEC-6 cells were seeded in 96-well plates at a density of 1×104 cells per well and incubated overnight at 37°C under 5% CO_2 atmosphere. The adherent cells were treated with different concentrations of CV (0, 1×10^4, 4×10^4, 9×10^4, 1.8×10^5, 3.5×10^5, 7×10^5 cells/mL), PA (0, 3.1, 6.3, 12.5, 25, 50, 100

μg/mL), blank gel (0, 4.7, 9.4, 18.8, 37.5, 75, 150 μg/mL) and CV@PA-gel (containing same concentrations of CV, PA and blank gel), respectively. After 24 h treatment, cells were incubated with CCK-8 reagent (Yeasen, Shanghai, China) for 2 h, and the absorbance at 450 nm was measured by a SpectraMax M5 plate reader (Molecular Devices, USA).

Fluorescence imaging

For in vitro fluorescence imaging, CV and CV@PA-gel samples were placed in the tubes and photographed by an IVIS Lumina LT Series III (PerkinElmer, USA) with the excitation (Ex) of 605 nm and emission (Em) of Cy5.5. For in vivo fluorescence imaging, male Balb/c nude mice (6 weeks, n = 3) were fasted overnight and were intragastrical injected with 300 μL of CV (2.8×10^7 cells/mL) and CV@PA-gel (CV = 2.8×10^7 cells/mL, PA = 4 mg/mL), respectively. At different time points before and after administration (0, 0.5, 1, 2, 4, 6, 8, 12, 24 h), mice were anesthetized with isoflurane gas and photographed by IVIS Lumina LT Series III. For ex vivo fluorescence imaging, male Balb/c nude mice (6 weeks, n = 3) were fasted overnight and were intragastrical injected with 300 μL of CV and CV@PA-gel, respectively. At different time points before and after administration (0, 0.5, 1, 2, 4, 6, 8, 12, 24 h), mice were euthanized and the major organs of mice, including heart, liver, spleen, lung, kidney and gastrointestinal tract, were collected and photographed by IVIS Lumina LT Series III.

Preliminary toxicity analysis

Male C57BL/6J mice (6 weeks) were randomly divided into four groups (n = 6): 1, Control; 2, PA; 3, CV; and 4, CV@PA-gel. During the treatment, mice were intragastrical administered with 300 μL of PBS, PA (4 mg/mL), CV (2.8×10^7 cells/mL) and CV@PA-gel (CV = 2.8×10^7 cells/mL, PA = 4 mg/mL) every other day for 30 days. After treatment, mice were euthanized and the blood samples

of mice in each group were collected for blood routine (n = 3) and blood chemistry (n = 3) analysis. The major organs of mice, including the brain, heart, liver, spleen, lung, kidney and gastrointestinal tract, were collected and processed for H&E staining.

Synthesis and characterization of CV@PA-gel

CV samples were collected by centrifugation (4500 rpm, 10 min) and washed three times with PBS. 2% (w/v) CMCS was mixed with 2% (w/v) SA at a volume ratio of 1:1. 1 mL of Chlorella vulgaris (2.8×10^7 cells/mL) and 1 mL of paeoniflorin (4 mg/mL) were added into the 3 mL of above CMCS/SA solution. The mixture was then crosslinked with 0%, 0.01%, 0.02%, and 0.04% (w/v) genipin at 37°C for 12 h, respectively. To obtain hydrogels with certain fluidity and adhesion, 0.02% genipine was used to synthesize CV@PA-gel. The bright-field and fluorescence images of CV were obtained by an optical microscope (Zeiss, Germany). The morphology of CV, blank-gel and CV@PA-gel were characterized by a field emission scanning electron microscopy (SEM, Hitachi SU-70, Japan). Ultraviolet-visible (UV-Vis) spectra of CV, PA and CV@PA-gel were recorded with a UV-2600 spectrophotometer (Shimadzu, Japan). Fluorescence emission spectra of CV and CV@PA-gel were acquired with a RF-6000 fluorescence spectrophotometer (Shimadzu, Japan) with an excitation wavelength at 552 nm. Zeta potential of CV, PA, blank-gel and CV@PA-gel were measured using a Malvern Zetasizer Nano-ZS90 (Malvern, UK). Fourier transform infrared (FTIR) spectra of CV, PA, blank-gel and CV@PA-gel were obtained with a FTIR spectrophotometer (Shimadzu, Japan) with a scan range of 400-4000 cm-1.

Quantitative Real-time PCR

Trizol extracted the RNA from tissue from animals or cells, cDNA was obtained by reverse transcription according to the manufacturer's instruction. The cDNA

was used for the real-time PCR with the SYBR Green PCR kit to measure the mRNA expression of specific molecules. The procedure of quantitative PCR instrument was described in our previous study. Information regarding molecular selection and primers is shown in Table S4.

ELISA analysis

According to the manufacturer's instructions, the levels of C3, GFAP, LPS and LBP in human plasma were detected, as well as the levels of BDNF and LBP of hippocampal homogenate and plasma in mouse.

Primary neuron, microglia, and astrocyte cell cultures

Primary hippocampus neurons were prepared from E16.5 pups of C57BL/6J mice; The cell suspension was inoculated with 7×10^4 cells/cm2 in different size petri dishes pre-coated with poly D-lysine (10μg/mL) and cultured in neurobasal medium with 2.5% inactivated fetal bovine serum, 2% B27 and 1% glutamax, 4 h after inoculation, the culture medium was changed as neurobasal medium with 2% B27 and 1% glutamax.

Primary hippocampal microglial and astrocytes were prepared from neonatal mice of C57BL/6J wild-type from P0-P1. The cell suspensions were inoculated in T75 culture bottles and incubated in Dulbecco modified Eagle Medium (DMEM) supplemented with 20% fetal bovine serum (FBS). The culture medium was replaced for 24 h. After about 10 days, the mixed glial cell population was separated into astrocyte rich fractions by shaking for 12 h (37°C, 200 rpm).

The conditional medium from primary microglia treated with either PBS, LPS, or LPS+PA was collected, which defined MCM, and applied to primary astrocytes for 24 h. After that, the conditional medium was collected, defined as ACM, and applied to primary neurons, respectively, for 24 h, for neuronal morphology and neuroapoptosis assay.

Molecular Docking

The molecular docking study was performed to investigate the interactions between paeoniflorin (PA) and the key microglia-derived cytokines (IL-1α, TNF-α, and C1q). The three-dimensional crystal structures of the target proteins—IL-1α (PDB ID: 5UC6), TNF-α (PDB ID: 4TSV), and C1Qa (PDB ID: 5HKJ)—were obtained from the RCSB Protein Data Bank (http://www.rcsb.org/). The structure of PA was downloaded in SDF format from the PubChem database (https://pubchem.ncbi.nlm.nih.gov/, CID: 442534).

Protein structures were prepared using PyMOL (Version 2.6.0) by removing water molecules and original ligands. The binding pocket parameters for each protein were defined using the Getbox Plugin in PyMOL. The prepared protein and ligand files were then imported into AutoDock Tools 1.5.6 for adding polar hydrogens, assigning charges, and converting to PDBQT format.

Docking simulations were carried out using AutoDock Vina 1.1.2 with an exhaustiveness value set to 20. The conformation with the most favorable binding energy was selected for each complex. Results were visualized and analyzed using PyMOL 2.6.0.

Immunofluorescence

The brain sections were first progressively dewaxed in different concentrations of alcohol, washed by PBS, and blocked by 5% BSA in PBS for 1 h at room temperature. After washing by PBS, cell cultures on coverslips were 4% polyformaldehyde fixed for 15 min, and incubated with 0.1% Triton X-100 in PBS for 15 min, blocked by 5% BSA in PBS at 37°C for 30 min. Then incubated with primary antibodies overnight at 4°C. After washing by PBS, the slides were incubated with fluorescent-dye conjugated secondary antibodies. Primary antibodies used were shown in the Table S4.

Hematoxylin and Eosin Staining

Paraffin sections were dewaxed in xylene and graded ethanol solutions, followed by staining with Harris hematoxylin and eosin, respectively, according to the manufacturer's instructions. The sections were then dehydrated and mounted before being imaged using a high-resolution microscope (BX53, Olympus, Japan) for subsequent analysis.

Nissl Staining

Dewaxed hippocampal sections were stained with the Nissl stain solution (Toluidine blue method) following the manufacturer's instructions. The sections were hydrated, stained, differentiated, and mounted before being imaged using a digital trinocular camera microscope (BX53, Olympus) for subsequent analysis.

Transmission electron microscope (TEM)

After perfusion with 4% paraformaldehyde, the hippocampus and colon of mice were manually cut into about 1 mm3 tissue and quickly fixed in 2.5% glutaraldehyde buffer overnight at 4°C. The tissues were transferred into 4% osmium tetroxide with 3% potassium ferrocyanide in 0.1 M cacodylate buffer at 4°C for 1 h, then embedded in Epon 812 after dehydration. Samples were cut into sections (0.4 μm) using an ultramicrotome and moved to copper grids. After staining with 2% aqueous uranyl acetate and followed by Reynold's lead citrate, the images were captured by using transmission electron microscopy (Thermo Scientific Talos L120C).

TEER assay

Caco-2 cells were co-cultured with Raw 264.7 cells by Transwell culture dish, PBS or PA or CV@PA preincubation in the Raw 264.7 cells for 1 h then LPS was added. Continued TEER was measured at 0, 1, 3, 6, 12 h.

Glycolysis rate test

According to the manufacturer's instructions, the glycolysis rate was determined on a seahorse XFe24 analyzer (Agilent, #103344-100). Briefly, astrocytes ($4×10^4$/well) were inoculated in XFe24 microplate plates. Once the astrocytes had adhered, 150uL additional medium was added to each group: PBS, MCM(LPS), MCM(LPS+PA) and MCM(LPS)+PA, respectively, incubating overnight. Prior to the assay, washed with the assay medium (103575-100 Seahorse XF DMEM medium) containing pyruvate (#103578-100), glutamine (#103579-100) and glucose (#103577-100), incubated in CO2-free 37°C for 40 min-60 min. During the assay, basal glycolysis, compensatory glycolysis and ECAR were determined, respectively, after the injection of Rot/AA (0.5 μM) and 2-DG (50 mM) at the specified time points.

FD4 Permeability Assay

We used FITC-labeled Dextran (FD4) to determine intestinal permeability in mice. Accurately dissolve FD4 in PBS at a final concentration of 100 mg/mL. Before the experiment, the mice were deprived of water and food for 8 h. During the 4-hour period, the gavage dose of FD4 was 6uL/g. PBS by intragastric administration for blank control. After continuing the fast for 4 h, blood was routinely collected in an EDTA anticoagulant tube and centrifuged at 3000 centrifuges at 4°C for 15 min, avoiding light during the whole process. The standard curve is produced by diluting the remaining FD4 liquid gradient.

For FD4 assay from the cell line, 1 mg/mL FD4 was added to the parietal chamber, and culture medium was collected from the basal lateral chamber after 2 h. The fluorescence intensity of FITC was quantified using a fluorescence plate reader (BioTek, Winooski, USA) with an excitation wavelength of 492 nm and an emission wavelength of 520 nm.

Analysis of 16S rRNA gene in gut microbiota

Fecal microbial DNA was extracted from mouse fecal samples for subsequent 16S rRNA sequencing analysis (Qubit dsDNA HS Assay Kit). The microbial composition and biodiversity were assessed, with alpha diversity evaluated using the chao1 index and beta diversity visualized through principal coordinate analysis (PCoA) based on the Bray-Curtis distance. Additionally, specific Phylum and Genus were identified based on their relative abundances. LEfSe (Linear discriminant analysis Effect Size) analysis is mainly used to find species with significant abundance differences between different groups (biomarkers).

Non-targeted metabolomics

Following previously described protocols, metabolomics data analysis was carried out on the fecal samples of mice, based on the LC-MS/MS system quadrupole - electrostatic field orbit trap Orbitrap mass spectrometer (Thermo Fisher Scientific, USA). Metabolite data were log2-transformed for subsequent statistical analysis. Differential metabolites were identified based on a combination of fold change and VIP (Variable Importance in Projection) value. The KEGG database was employed for annotating the identified metabolites, as previously described.

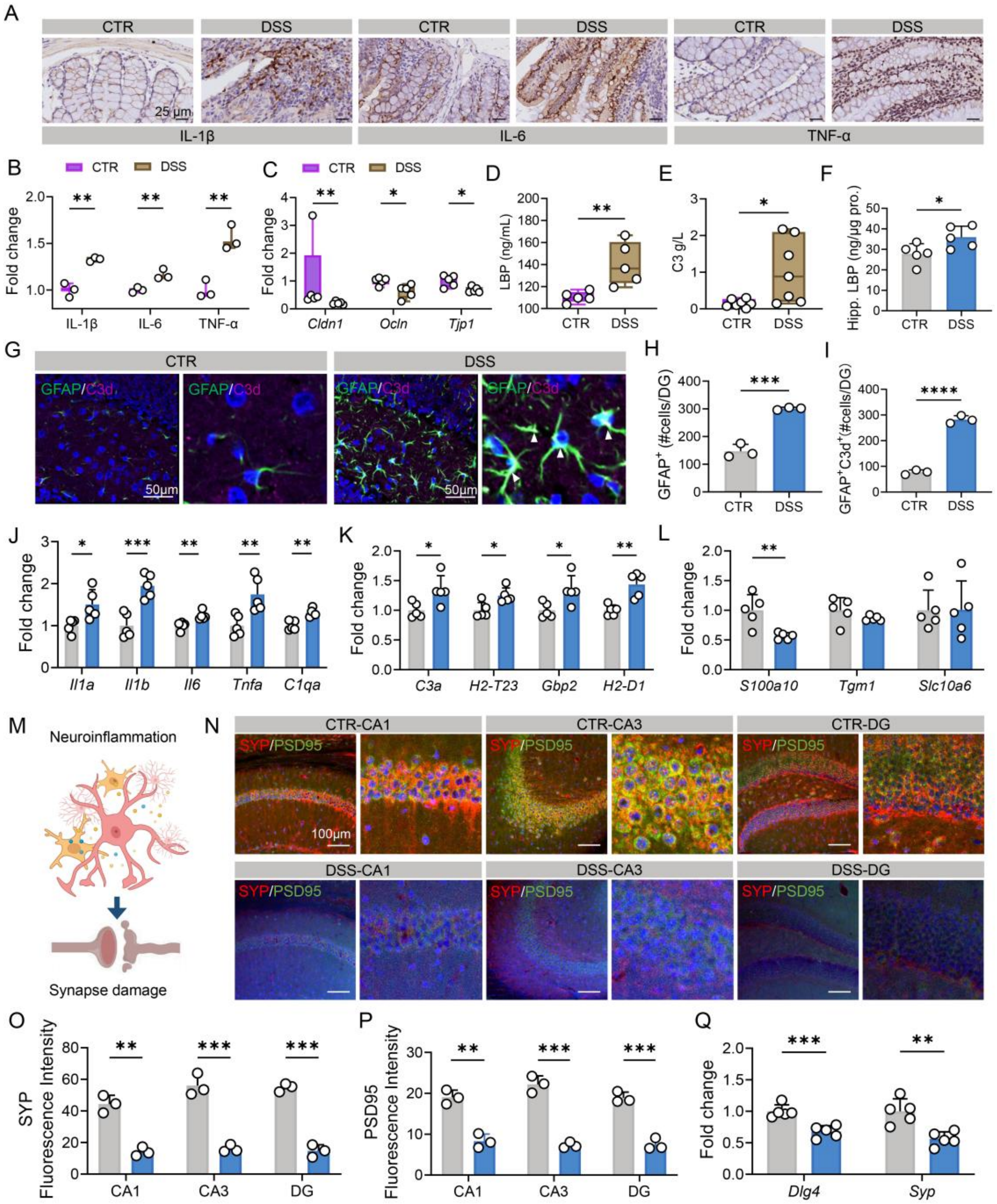

Figure S1. Effects on DSS-induced intestinal inflammation and cognitive dysfunction and synaptic impairment in mouse. (A) Immunohistochemical (IL-1β, IL-6 and TNF-α) staining of colon tissues in different groups. Scale bars, 25μm. (B) Quantitative results of relative positive intensities of TNF-α, IL-1β, and IL-6 of colon tissues in different groups (n=3). (C) mRNA expression of intestinal barrier-associated factors including *Ocln*, *Tjp1* and *Cldn1* in colon tissues of mice in different groups (n=5). (D-E) Plasma LBP (D, n=5) and C3 (E,

n=7) level in DSS and control (CTR) group. (F) hippocampus LBP level of in DSS and CTR group (n=5). (G) Representative microscopic fields of GFAP$^+$C3d$^+$ cells in the DG of the hippocampus of mice. Scale bars, 50 μm. (H-I) Quantification of GFAP$^+$ (H) and GFAP$^+$C3d$^+$ (I) cells in the DG of the hippocampus of mice (n=3). (J-L) Active microglia (*Il1a*, *Il1b*, *Il6*, *Tnfa* and *C1qa*) (J), A1-type (*C3a*, *H2-T23*, *Gbp2*, and *H2-D1*) (K) and A2-type (*S100a10*, *Tgm1*, and *Slc10a6*) (L) astrocyte marker-related molecules mRNA expression from the hippocampus in DSS and CTR group (n=5). (M) Neuroinflammation induces impaired synaptic plasticity. (N-P) Representative microscopic fields (N) and mean fluorescence intensity of SYP (O) and PSD-95 (P) in the CA1, CA3 and DG of the hippocampus in different groups (n=3). Scale bars, 100 μm. (Q) mRNA expression of synapse-associated protein (Dlg4 and Syp) from the hippocampus in DSS and CTR group (n=5). The significance of difference of (B), *Ocln* and *Tjp1* in (C), (D-F), (H-L) and (O-Q) was determined by unpaired *t*-test while the significance of difference of Cldn1 in (C) was determined by Mann-Whitney test. ns, no significance $p \geq 0.05$; *$p < 0.05$, **$p < 0.01$, and ***$p < 0.001$, ****$p < 0.0001$.

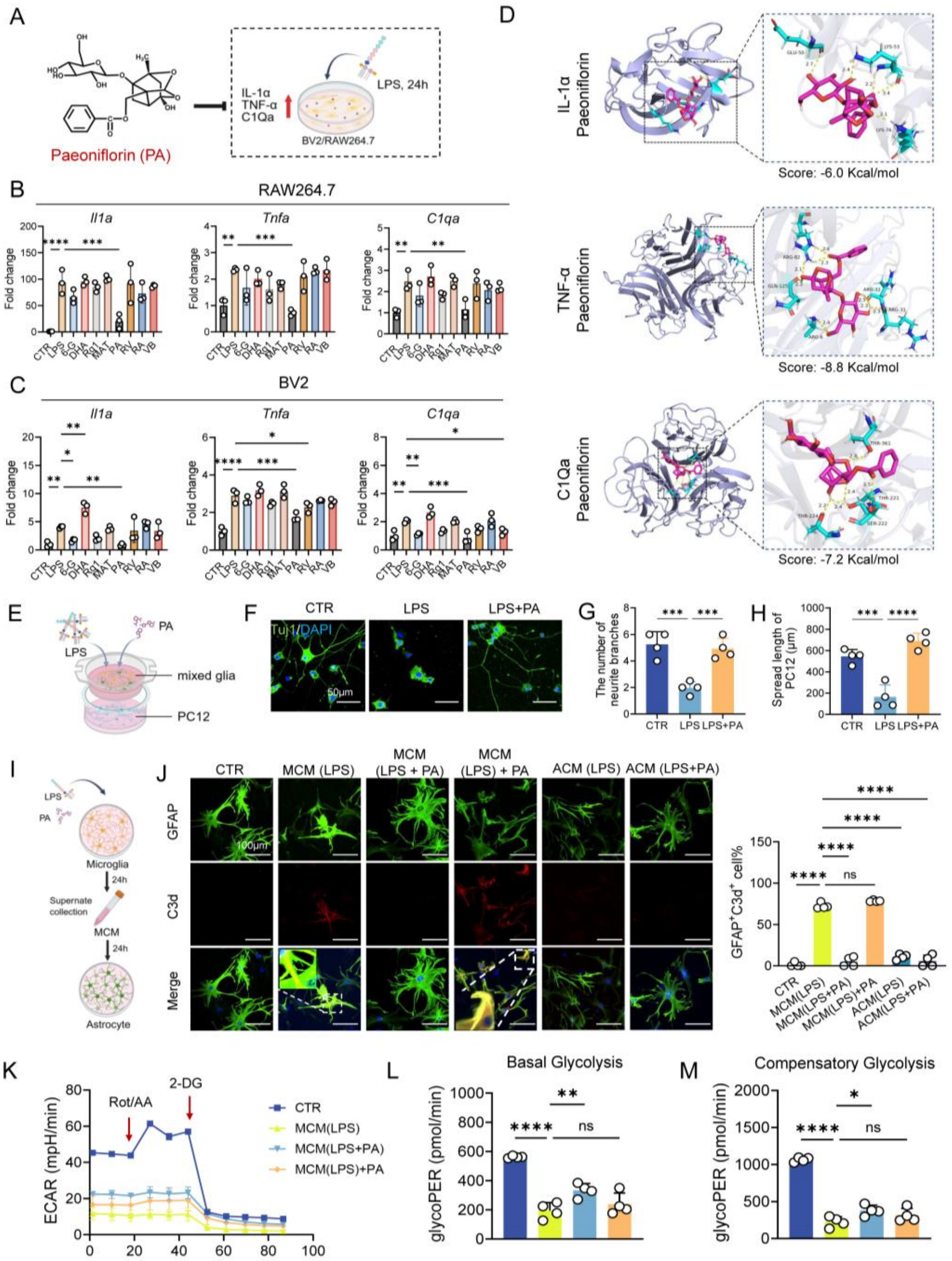

Figure S2. Inhibition of the target of A1 reactive astrocytes and the neurogenesis by paeoniflorin (PA). (A) PA inhibits the increase in IL-1α, TNF-α and C1Qa secretion of microglia induced by LPS. (B-C) mRNA expression of Il1a, Tnfa and C1qa in RAW264.7 (C) and BV2 (D) in different groups (n=3). (D) Molecular docking visualization of PA binding to IL-1α, TNF-α and C1Qa. (E) Schematic diagram showing the co-culture if mixed glia and PC12. (F-G) Representative microscopic fields (F) and the quantification of neurite branches (G) and speed length (H) in PC12 cell lines in different groups (n=4). (I)

Schematic diagram showing the collection of astrocyte and neuron samples after treatment with microglia-conditioned medium (MCM) or astrocyte-conditioned medium (ACM), considering the regeneration of neurons by PA-treated microglia or astrocytes. (J) Representative microscopic fields and quantification of GFAP+C3d+ cells in astrocytes (n=4). Scale bar, 100μm. (K-M) The glycolysis rate test. Changes of extracellular acidification rate (ECAR) (K), basal glycolysis (L) and compensatory glycolysis (M) in astrocytes (n=4). The significance of difference of (B-C), (G-H), (J) and (L-M) was determined by one-way ANOVA with Dunnett's post hoc test. ns, no significance $p \geq 0.05$; $*p < 0.05$; $**p < 0.01$; $***p < 0.001$; $****p < 0.0001$.

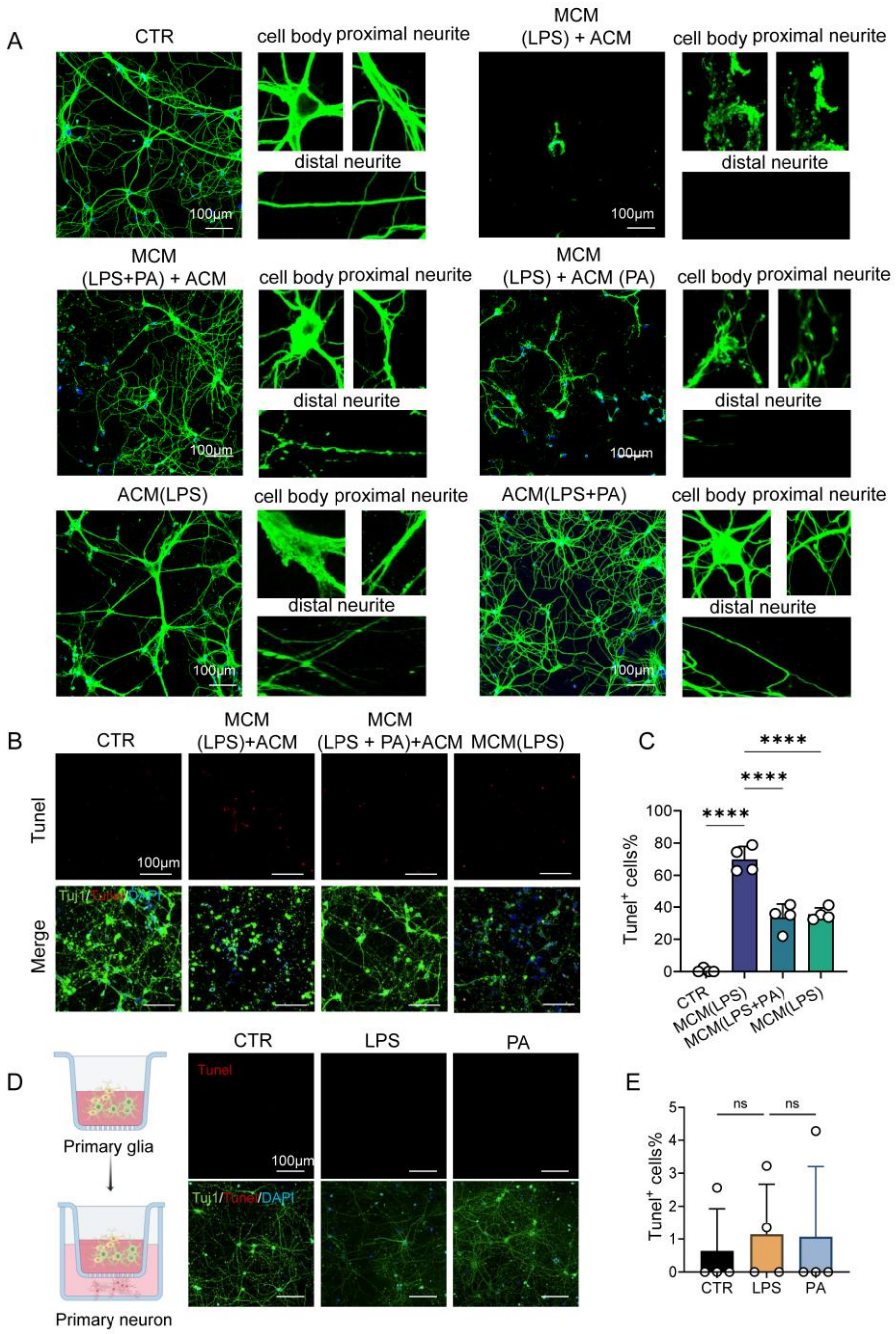

Figure S3. PA inhibits A1 reactive astrocyte activation. (A) Morphological changes of hippocampal primary neurons in different groups. Scale bar, 100μm. (B-E) Representative microscopic fields (B, D) and quantifications (C, E) of Tunel+ cells in hippocampal primary neurons (n=4). Scale bar, 100μm. The significance of difference of (C) and (E) was determined by one-way ANOVA with Dunnett's post hoc test. Not significant (ns) $p \geq 0.05$, *$p < 0.05$, **$p < 0.01$, ***$p < 0.001$ and ****$p < 0.0001$.

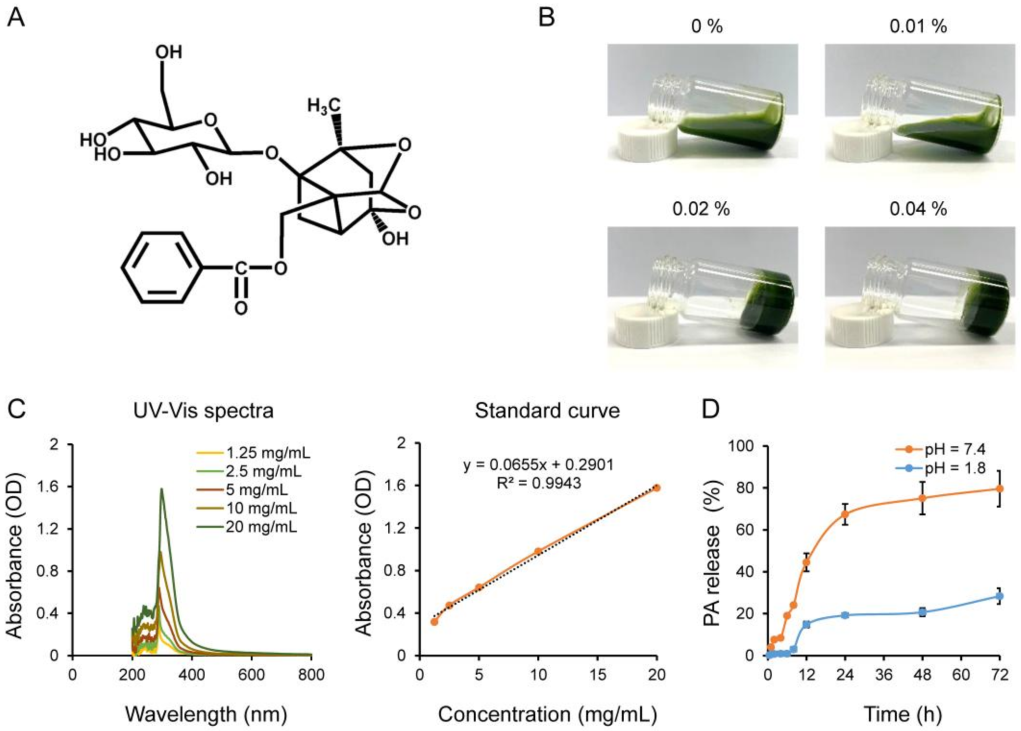

Figure S4. (A) Chemical structure of paeoniflorin. (B) Photographs of CV@PA-gel formed at different final concentrations of genipin (0, 0.01, 0.02, and 0.04%). (C) UV-Vis spectra of paeoniflorin in PBS solutions with different concentrations (1.25, 2.5, 5, 10, and 20 mg/mL). Standard curve of paeoniflorin in PBS measuring at 413 nm. PBS, phosphate buffer saline. (D) In vitro release profiles of PA from the PA-loaded hydrogel without microalgae under different pH conditions.

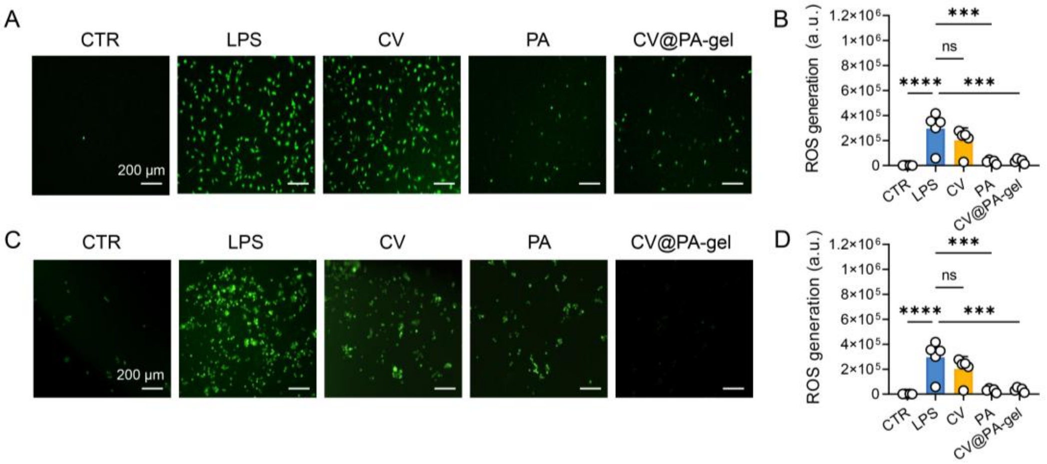

Figure S5. In vitro cytocompatibility of CV, PA, Blank-gel, and CV@PA-gel in IEC-6 and RAW264.7 cells. (A-B) Representative images (A) and quantification (B) of ROS generation in IEC-6 cells after different treatments (n=5). Scale bar, 200 μm. (C-D) Representative images (C) and quantification (D) of ROS generation in RAW264.7 cells after different treatments (n=5). Scale bar, 200 μm.

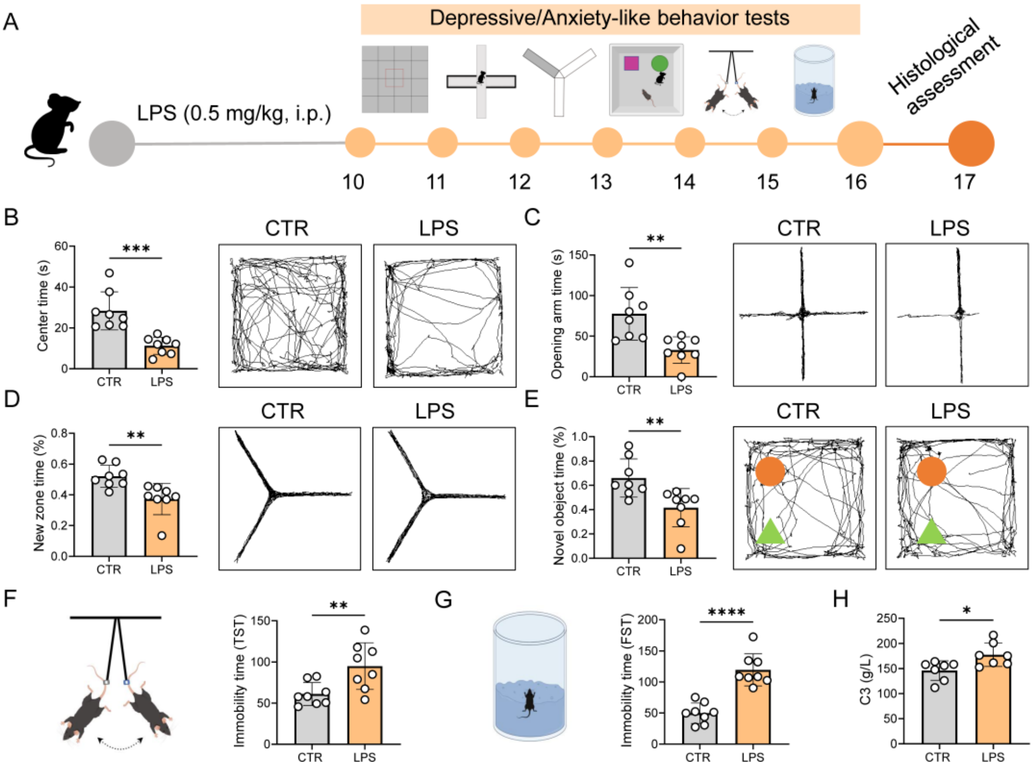

Figure S6. Systemic endotoxemia drives complement-mediated neuropsychiatric deficits. (A) Schematic illustration of the construction of the LPS-induced depressed model. (B-G) The center time in OFT (B), opening arm time in EPM (C), new zone time in Y-maze (D), novel object time in NOR (E), the immobility time in TST (F) and FST (G) in different groups (n=8). (H) Plasma C3 level in different groups (n=7). The significance of difference of (B-G) was determined by unpaired *t*-test. ns, no significance $p \geq 0.05$; *$p < 0.05$; **$p < 0.01$; ***$p < 0.001$; ****$p < 0.0001$.

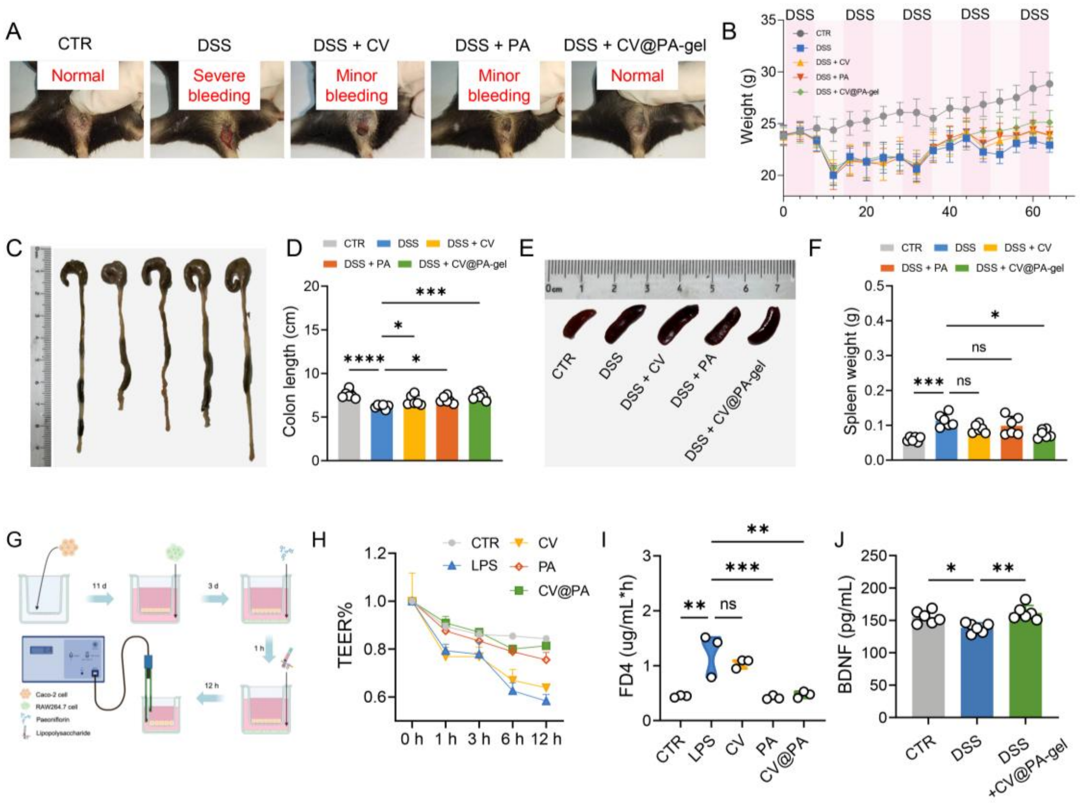

Figure S7. CV@PA-gel treatment alleviated DSS-induced enteritis characterization. (A) Photographs of rectal bleeding of mice in different groups during treatment. (B) Body weight of different groups during the treatment (n=11). (C-D) Photograph (C) and quantitative results (D) of colon length in different groups after treatment in different groups (n=6). (E-F) Photographs (E) and quantitative results (F) of the weight of spleen tissues of mice in different groups (n=6). (G) Caco-2 cells were co-cultured with Raw 264.7 cells by trans well culture dish, PBS or PA or CV@PA preincubation in the Raw 264.7 cells for 1 hour then LPS was added. (H) Continued TEER was measured at 0, 1,3, 6, 12 h. (I) FD4 flux in Caco-2 cell monolayers after different treatments (n=3). (J) Hippocampal brain-derived neurotrophic factor (BDNF) in CTR, DSS and DSS+CV@PA-gel group (n=6). The significance of difference of (D) and (I-J) was determined by one-way ANOVA with Dunnett's post hoc test while the significance of difference of (F) was determined by Kruskal-Wallis test. ns, no significance $p \geq 0.05$; *$p < 0.05$; **$p < 0.01$; ***$p < 0.001$; ****$p < 0.0001$.

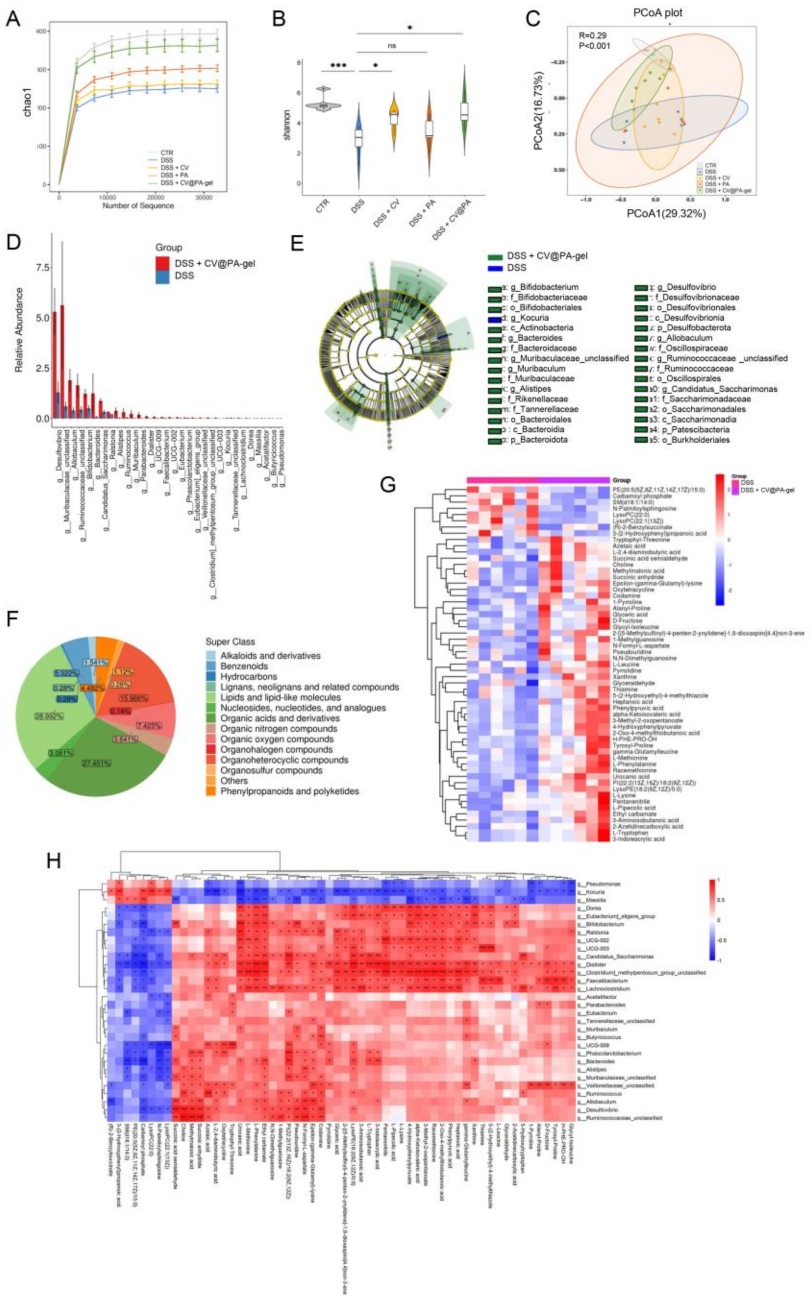

Figure S8. Metagenomic bioinformatic analysis and fecal metabolite alteration in different groups. (A-B) 16S ribosomal RNA gene sequencing of fecal samples showed bacterial diversity (n=6, biologically independent animals). (C) Principal coordinates analysis (PCoA) maps showed gut microbiota characteristics of different groups. (D) Relative abundance of microbiota at the genus levels. (E) The volcano map exhibits the difference in metabolites between the DSS and CV@PA-gel treatment group. (F) Pie chart

of differential metabolism. (G) Differential metabolites heat map between the DSS and CV@PA-gel group. (H) Correlation between differential metabolites and differential bacteria. Not significant (ns) $p \geq 0.05$, *$p < 0.05$, **$p < 0.01$, ***$p < 0.001$ and ****$p < 0.0001$.

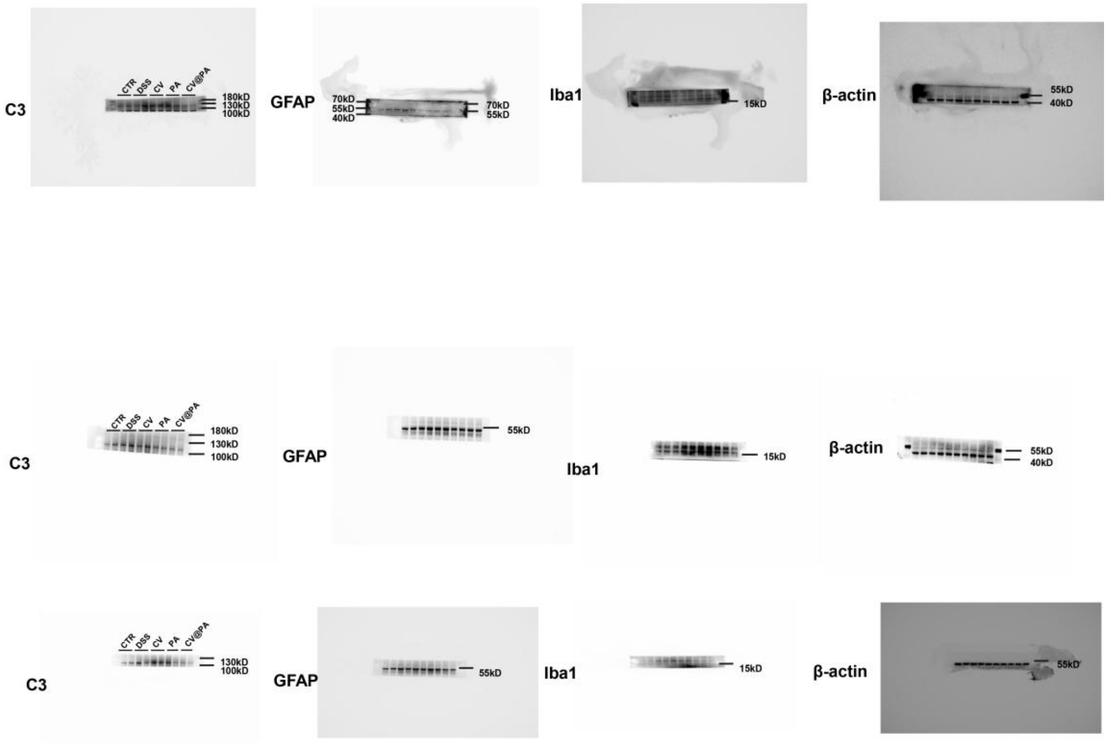

Figure S9. Raw images of western blots in Figure 6I (Protein Ladder 26616 produced by Thermo Fisher Scientific).

Tables S1. Clinical characteristics of study cohorts, related to Figure 1.

Measure	CTR (N=17)	IBD (N=19)	t/χ2/F	*p* value
Age (y)	35.76±8.408	35.58±12.67	0.05116	0.9595
Sex（male/female）	9/8	12/7	0.6207	0.5348
Education years	14.76±1.562	14.47±2.170	0.4568	0.6507
Marriage (unmarried/married)	9/8	10/9	0.8396	0.3595
HADS-A	1.059±0.8993	6.895±3.446	6.769	＜0.0001
HADS-D	1.235±0.9034	5.526±3.533	4.860	＜0.0001

Tables S2. The ability of different compounds to improve the activation of microglia induced by LPS.

Compounds	Pharmacological activities	A1 Astrocyte Inducers reduction ability (%) (Compared to LPS)					
		RAW264.7			BV2		
		Il1a	***Tnfa***	***C1qa***	***Il1a***	***Tnfa***	***C1qa***
6-Gingerol (6-G)	anti-inflammatory, gut microbiome modulation [16]	29.4	27.1	28.9	57.8	9.10	44.5
Dihydroartemisinin (DHA)	anti-inflammatory and colitis alleviation[16]	-1.70	-9.40	14.8	-86.1	-9.60	-30.3
Ginsenoside Rg1 (Rg1)	anti-inflammatory, gut microbiome modulation, synaptic plasticity regulation, anti-depressant [16]	9.79	26.4	31.7	48.7	15.1	34.3
Matrine (MAT)	anti-inflammatory, colitis alleviation and anti-depressant [16]	-7.30	-1.10	24.0	7.24	-8.00	0.230
Paeoniflorin (PA)	anti-inflammatory neuroprotection, and anti-depressant [16]	78.2	53.4	69.1	77.6	39.5	58.0
Resveratrol (RV)	anti-inflammatory, neuroprotection and psychiatric disorders	1.67	3.80	10.2	15.4	21.3	27.1

	improvement [16]						
Rosmarinic Acid (RA)	anti-inflammatory and anti-depressant [16]	22.4	16.5	1.81	-11.9	8.85	-4.30
Verbascoside (VB)	neuroprotection and anti-depressant [16]	7.66	12.1	0.630	16.6	12.0	38.1

Table S3. Histopathological score of intestinal damage.

**Score**	**Severity of inflammation**	**The extent of inflammation**	**Crypt damage**
0	Not present	Not present	Not present
1	Mild inflammation	Mucosa diffuse infiltration	Mild accumulation
2	Moderate inflammation	Mucosa and submucosa diffuse infiltration	Moderate accumulation
3	Severe inflammation	Transmural diffuse infiltration	Severe accumulation

Tables S4. The list of primers used in qRT-PCR.

Gene	Primer sequences (Forward)	Primer sequences (Reverse)
Cldn1	GTGCACTTGGGAAAGCTGTG	CTTTGCGAAACGCAGGACAT
Ocln	TCCACTTGGTGGTTTGCTGA	TCCACTTGGTGGTTTGCTGA
Tjp1	GCCGCTAAGAGCACAGCAA	GCCCTCCTTTTAACACATCAGA
Dlg4	AGTTGCAGGTGAACGGAACA	TCACCGATGTGTGGGTTGTC
Syp	GGGCCAATGATGGACTTCCT	GCCTGTCTCCTTGAACACGA
Il1a	GGAGGGCCATGCGAATCTAA	AAGGTGCTGATCTGGGTTGG
Il1b	TGCCACCTTTTGACAGTGATG	TGATGTGCTGCTGCGAGATT
Il6	TCCTACCCCAATTTCCAATGCT	CGCACTAGGTTTGCCGAGTA
Tnf	GGCAGTTAGGCATGGGATGA	TCCACTTGGTGGTTTGCTGA
C1qa	TTCCTCATTTTCCCCTCGGC	GACACAGACGGGGATCGTTT
C3	ATAAAGAGCCAGCGGCTACA	CAGCCGTAGGACATTGGGAG
H2-T23	AGTAAACCTGAGGACCCTGC	TCTGTGAGGCTATGTCATTCGC
Gbp2	CAGCCGTAGGACATTGGGAG	AACATAGGTCTGCACCAGGC
H2-D1	TGGTGCTGCAGAGCATTACA	CACCACAGATGCCCACTTCT
S100a10	TCTGTGAGGCTATGTCATTCGC	ATTCCTCAAGTGACCCCGTG
Tgm1	GGCAGGTACTAGCCAAGCAA	GTGCACTTGGGAAAGCTGTG
Slc10a6	GGAGGGCCATGCGAATCTAA	GGAGGGCCATGCGAATCTAA
Nos2	TCCAGAGGGGAGTAGACCAAG	AGAAATGAGGGCACCTAGCC
Rela	CCTGCAGGGTCACTCGATTT	TCAGAACCAAGAAGGACGCC
Actb	TGCGGGATGGTCAGTTAAGAG	CCTTCTGACCCATTCCCACC

Cldn1, claudin 1; Ocln, occludin; Tjp1, tight junction protein 1, also ZO-1; Dlg4, discs large MAGUK scaffold protein 4, also PSD95; Syp, synaptophysin; Il1a, interleukin 1 alpha; Il1b, interleukin 1 beta; Il6, interleukin 6; Tnf, tumor necrosis factor; C1qa, complement component 1, q subcomponent, alpha polypeptide; C3, complement C3; H2-T23, histocompatibility 2, T region locus 23; Gbp2, guanylate binding protein 2; H2-D1, histocompatibility 2, D region locus 1; S100a10, S100 calcium binding protein A10; Tgm1, transglutaminase 1, K

polypeptide; Slc10a6, solute carrier family 10, member 6; Nos2, nitric oxide synthase 2, inducible, also iNOS; Rela , v-rel reticuloendotheliosis viral oncogene homolog A, also p65; Actb, actin, beta.

Tables S5. Key Resources Table.

**REAGENT or RESOURCE**	**SOURCE**	**IDENTIFIER**
**Antibodies**		
IL-1β	Abcam	# ab283818
IL-6	Wanleibio	# WL02841
TNF-α	Abcam	# ab183218
ZO-1	Abcam	# ab221547
Occludin	Abcam	# ab216327
Claudin-1	NOVUS	# H00009076-M01
GFAP	Abcam	# ab7260
SOX2	Abcam	# ab93689
SYP	Proteintech	# 67864-1-Ig
PSD95	Proteintech	# 20665-1-AP
C3	Proteintech	# 21337-1-AP
C3d	R&D System	# AF2655
Doublecortion	Abcam	# ab18723
IBA1	Abcam	# ab178846
β-Actin	Cell Signaling Technology	# 3700S
Tuj1	Proteintech	# 66375-1-Ig
**Chemicals and commercial kits**		
LPS	Sigma	# L4130
B27	Gibco	# 17504044
CELLSAVING	New Cell & Molecular Biotech	# C40100
FITC-Dextran 4	Sigma	# 60842-46-8

Halt™ Protease and Phosphatase Inhibitor Single-Use Cocktail	Thermo Scientific	# 78446
Dextran Sulfate Sodium Salt	Yeasen	# 60316ES76
Human endotoxin binding protein (LBP) ELISA kit	Abcam	# ab279407
Human lipopolysaccharide/endotoxin (LPS) ELISA kit	AiFang	# AF1302-A
Human glial fibrillary acidic protein (GFAP) ELISA kit	Abcam	# ab223867
Mouse Lipopolysaccharide binding protein ELISA Kit	Abcam	# ab269542
Mouse Brain derived neurotrophic factor ELISA Kit	AiFang	# AF2204-
Complement C3 content (immunoturbidimetry) detection kit	Jianglai biology	# JL-T2636
Cytosine Arabinoside (Ara-C)	MCE	# HY-13605
Beta-NGF	MCE	#HYP700162AF
Paeoniflorin	RHAWN	# R002156
Super FastPure Cell RNA Isolation Kit	Vazyme	# RC102-01
HiScript IV All-in-One Ultra RT SuperMix for qPCR	Vazyme	# R433-01
TransStart® Green qPCR SuperMix	TransGen	# AQ101-01
2-Hour Rapid Western Blot Ready-to-Use Complete Workflow Kit	EnkiLife	# RA10020
ECL Enhanced Kit	Seven (Beijing, China)	# SW181-02
Reactive Oxygen Species Assay Kit	Yeasen	# 50101ES01
TUNEL Apoptosis Detection Kit	Yeasen	# 40307ES60

Special fetal bovine serum	BaiDi Biotechnology Co., Ltd.(BDBIO)	# F801-500
Dulbecco's modified eagle medium	BaiDi Biotechnology Co., Ltd.(BDBIO)	# L100-500
Seahorse XF Glycolytic Rate Assay Starter Pack	Agilent	# 103344-100
